# Visible and Near‐Infrared Photothermal Catalyzed Hydrogenation of Gaseous CO_2_ over Nanostructured Pd@Nb_2_O_5_

**DOI:** 10.1002/advs.201600189

**Published:** 2016-07-05

**Authors:** Jia Jia, Paul G. O'Brien, Le He, Qiao Qiao, Teng Fei, Laura M. Reyes, Timothy E. Burrow, Yuchan Dong, Kristine Liao, Maria Varela, Stephen J. Pennycook, Mohamad Hmadeh, Amr S. Helmy, Nazir P. Kherani, Doug D. Perovic, Geoffrey A. Ozin

**Affiliations:** ^1^ Department of Materials Science & EngineeringUniversity of Toronto184 College StreetTorontoOntarioM5S 3E4Canada; ^2^ Materials Chemistry and Nanochemistry Research GroupSolar Fuels ClusterDepartment of ChemistryUniversity of Toronto80 St. George StreetTorontoOntarioM5S 3H6Canada; ^3^ Institute of Functional Nano and Soft Materials (FUNSOM)Jiangsu Key Laboratory of Carbon‐Based Functional Materials and Devices, and Collaborative Innovation Center of SuzhouNano Science and TechnologySoochow University215123SuzhouJiangsuChina; ^4^ Materials Science and Technology DepartmentOak Ridge National LaboratoryOak RidgeTN37831USA; ^5^ GFMC & Instituto PluridisciplinarUniversidad Complutense de MadridMadrid28040Spain; ^6^ National University of SingaporeDepartment of Materials Science and EngineeringBlock EA #07‐14, 9 Engineering Drive 1117575SingaporeSingapore; ^7^ Department of ChemistryAmerican University of BeirutBeirut11‐0236Lebanon; ^8^ Department of Electrical and Computing EngineeringUniversity of Toronto10 King's College RoadTorontoOntarioM5S 3G4Canada

**Keywords:** carbon dioxide, palladium catalysts, photothermal catalysis, niobium oxide nanostructure, solar fuels

## Abstract

The reverse water gas shift (RWGS) reaction driven by Nb_2_O_5_ nanorod‐supported Pd nanocrystals without external heating using visible and near infrared (NIR) light is demonstrated. By measuring the dependence of the RWGS reaction rates on the intensity and spectral power distribution of filtered light incident onto the nanostructured Pd@Nb_2_O_5_ catalyst, it is determined that the RWGS reaction is activated photothermally. That is the RWGS reaction is initiated by heat generated from thermalization of charge carriers in the Pd nanocrystals that are excited by interband and intraband absorption of visible and NIR light. Taking advantage of this photothermal effect, a visible and NIR responsive Pd@Nb_2_O_5_ hybrid catalyst that efficiently hydrogenates CO_2_ to CO at an impressive rate as high as 1.8 mmol gcat^−1^ h^−1^ is developed. The mechanism of this photothermal reaction involves H_2_ dissociation on Pd nanocrystals and subsequent spillover of H to the Nb_2_O_5_ nanorods whereupon adsorbed CO_2_ is hydrogenated to CO. This work represents a significant enhancement in our understanding of the underlying mechanism of photothermally driven CO_2_ reduction and will help guide the way toward the development of highly efficient catalysts that exploit the full solar spectrum to convert gas‐phase CO_2_ to valuable chemicals and fuels.

## 1 Introduction

Solar fuels are attracting increasing attention owing to their potential of being a viable alternative to fossil fuels. One appealing route to the production of solar fuels is to use renewable sources of hydrogen to reduce the greenhouse gas CO_2_ to value‐added chemicals and fuels.^1^ If realized in practice at a technologically significant efficiency, economically competitive cost and industrially relevant scale, this could simultaneously address several significant global challenges: climate change, renewable energy, and protection of the environment.^2^, ^3^, ^4^

The activity of various photocatalyts toward the light‐assisted reduction of CO_2_ to useful fuels has been studied.^3^, ^4^, ^5^, ^6^ Among different approaches that convert CO_2_ into useful products, gas‐phase light‐assisted reduction of CO_2_ is a practical option, since gas‐phase processes can be easily scaled and integrated with existing chemical and petrochemical industry infrastructure.^7^ More recently, a photothermal approach in which gas‐phase light‐assisted hydrogenation of CO_2_ to useful fuels over supported metallic catalysts, which are traditionally known to be activated thermally at elevated temperatures, has been investigated. In this context, the photothermal approach to the catalytic hydrogenation of CO_2_ has emerged with reported conversion rates as high as mmol g_cat_^−1^ h^−1^ to mol g_cat_^−1^ h^−1^.^8^, ^9^ This tactic exploits the transformation of light into heat, generating high local temperatures in nanostructured catalysts that drive CO_2_ reduction reactions. Today however, the mechanism of photothermal catalysis remains unclear. By identifying the key factors which underpin the photothermal effect that promotes catalytic CO_2_ reduction reactions, it will prove possible to design and make nanoscale catalysts with structural, chemical, and physical properties that serve to maximize the rate of reduction of gaseous CO_2_ to chemicals and fuels, using both the heat and light from sunlight.

For the case of metal nanocrystals, the photothermal effect can arise in different ways that include surface plasmon resonance (SPR) and nonradiative relaxation, and intraband or interband nonradiative relaxation of photoexcited charge carriers. The SPR effect occurs when absorbed radiant energy is stored in the collective resonant oscillations of conduction electrons.^6^, ^10^, ^11^, ^12^ On the other hand, interband and intraband absorption commonly occur in transition metals when electrons are photoexcited to higher energetic states within the same electronic band or between different bands, respectively.^13^, ^14^ For the case of Pd nanocrystals below 10 nm in size, the SPR is around 200–250 nm and these nanocrystals exhibit strong ultraviolet (UV) light absorption.^15^ Furthermore, visible and near infrared (NIR) light absorption in these Pd nanocrystals is dominated by interband electron transitions (between the *d* band and *s*‐*p* conduction band) and intraband transitions (between filled and empty states in the d and s–p bands).^10^, ^11^, ^14^, ^16^

The catalyst support may also influence the photothermal effect. Recent efforts toward the development of photothermal catalysts for gas‐phase CO_2_ reduction have focused on metal nanocrystals dispersed on insulating metal oxide supports.^9^ Photothermal catalysts based on metal nanocrystals deposited on metal oxide semiconductor supports, especially reducible metal oxide supports, have received much less attention. Intuitively, such photoactive reducible semiconductor supports may further accelerate the catalytic reactions through different mechanisms. For example, defects in metal oxide semiconductors, such as oxygen vacancies and reduced or coordinately unsaturated metal sites, can promote the affinity of CO_2_ to the surface of the catalysts, prolong photogenerated charge‐carrier lifetimes and enhance the reactivity of CO_2_.^4^, ^17^, ^18^, ^19^

Herein we report the reverse water gas shift (RWGS) reaction (CO_2_ + H_2_ **→** CO + H_2_O) driven with visible and near‐infrared photons over hybrid catalysts composed of palladium nanocrystals dispersed on Nb_2_O_5_ nanorods denoted Pd@Nb_2_O_5_. Relevant prior work on Pd nanocrystals includes electrocatalytic,^20^ photocatalytic,^21^ and thermocatalytic hydrogenation of CO_2_,^22^, ^23^, ^24^ CO_2_ photothermal methanation,^9^ as well as photothermal hydrogenation of styrene.^25^ The thermally driven systems promote CO_2_ hydrogenation at high rates but require high operating temperatures (523 K–900 K) and high pressures.^22^, ^23^, ^24^

In this study, we use nanostructured Pd@Nb_2_O_5_ as a model hybrid catalyst system to demonstrate the potential of driving the RWGS reaction using light, without providing external heating to the catalyst. Specifically, we observe CO_2_‐to‐CO hydrogenation rates as high as 4.9 mmol g_cat_^−1^ h^−1^ over these hybrid Pd@Nb_2_O_5_ catalysts when subjected to light emitted from a Xe lamp at an incident intensity of 25 kW m^−2^. This rate is only about two orders of magnitude less than a rate considered to be of technological significance. Notably, the CO_2_ hydrogenation reaction proceeded at a rate of 1.8 mmol g_cat_^−1^ h^−1^ using light in the visible and NIR spectral region. We also attempt to pinpoint the key factors responsible for the photothermal hydrogenation of CO_2_‐to‐CO over Pd@Nb_2_O_5_ by investigating the reaction rate dependence on the spectral power distribution and intensity of the incident light. Furthermore, we utilize variable temperature Raman spectroscopy measurements to investigate the photothermal effect on the local temperature of individual and assemblies of the nanocomponents that comprise our Pd@Nb_2_O_5_ hybrid catalysts. Additionally, we have observed an enhancement of the CO_2_ conversion rate that is connected to the in situ generation of Nb^4+^ and/or O vacancy defects. This effect more than doubles the RWGS rate.

## 2 Results and Discussion

### 2.1 Characterization of Nanostructured Pd@Nb_2_O_5_

**Figure 1:**
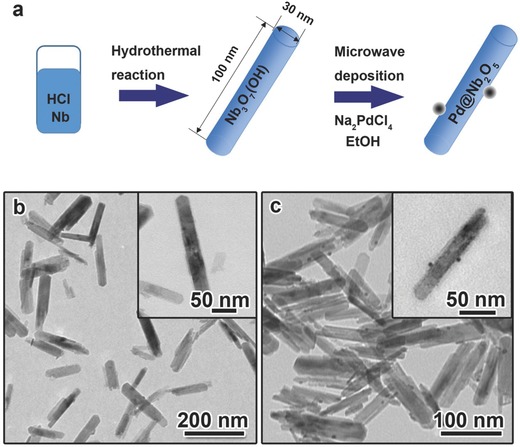
Synthesis, morphology and structure of Nb_3_O_7_(OH) nanorods and Pd@Nb_2_O_5_ nanocrystal–nanorod samples. a) Scheme of the synthesis of Nb_3_O_7_(OH) and Pd@Nb_2_O_5_ nanorod samples. b) TEM images of Nb_3_O_7_(OH) nanorod sample. c) TEM images of Pd@Nb_2_O_5_ nanocrystal–nanorod sample.

Nb_3_O_7_(OH) was prepared via hydrothermal synthesis as described in the experimental section and illustrated in **Figure** **1**a. TEM images of the sample are shown in Figure 1b, which exhibit nanorod shapes, with an average length and width of 100 nm and 30 nm, respectively. The nanorod‐shaped morphologies of the as‐synthesized Nb_3_O_7_(OH) reflect the orthorhombic crystal structure and underlying symmetry of the unit cell.^26^ Aberration corrected scanning transmission electron microscopy (STEM) was used to characterize the atomic structure of the Nb_3_O_7_(OH) nanorods. Figure S1a (Supporting Information) is a high angle annular dark field (HAADF) image of the Nb_3_O_7_(OH) nanorods at low magnification. Figure S1b (Supporting Information) shows an atomic resolution HAADF image from a nanorod with its [001] direction parallel to the electron beam. The FFT of the image is shown in Figure S1c (Supporting Information), the (2 0 0), (1 1 0), and (0 2 0) spots can be clearly resolved and the viewing direction is confirmed to be [001]. From both the image and the FFT, [010] is found to be the preferred growth direction of the Nb_3_O_7_(OH) nanorods. Figure S1d (Supporting Information) is an atomic model of Nb_3_O_7_(OH) marked by the red rectangle in Figure S1b (Supporting Information). The dotted blue rectangle in Figure S1d (Supporting Information) indicates one unit cell.

In the literature, Pd nanocrystals are typically loaded onto catalyst supports using an impregnation method wherein the supports are immersed in a palladium precursor solution prior to being thermally treated. However, in this work we used a straightforward microwave‐assisted hydrothermal reduction to decorate the Nb_3_O_7_(OH) nanorods with Pd nanoparticles (Figure 1a). With the use of ethanol as a mild reducing agent, the reaction rate could be well controlled to minimize the self‐nucleation of Pd nanocrystals unattached to nanorods. The existence of surface hydroxide groups on the Nb_3_O_7_(OH) nanorods is believed to provide binding sites for Pd nanocrystals. Therefore, the hybrid structures were obtained devoid of free Pd nanocrystals, as seen in the TEM image shown in Figure 1c.

To transform Nb_3_O_7_(OH) nanorods to Nb_2_O_5_ nanorods, we pre‐treated Pd@Nb_3_O_7_(OH) at 120 °C for 24 h. The PXRD patterns in Figure S2 (Supporting Information) show that before the pre‐treatment the sample consists of orthorhombic Nb_3_O_7_(OH), while the treated sample underwent a structural transformation from orthorhombic Nb_3_O_7_(OH) to monoclinic Nb_2_O_5_. Due to the low Pd loading (0.5 wt%) and the small size of the nanocrystals, diffraction peaks associated with palladium species were not observed. As shown in the TEM images of pure Nb_3_O_7_(OH) nanorods before the pre‐treatment (Figure S3a, Supporting Information) and Nb_2_O_5_ nanorods after the pre‐treatment (Figure S3b, Supporting Information), the nanorod morphology is retained. The TEM image in Figure 1c clearly depicts the heterojunction between the Pd nanocrystals and Nb_2_O_5_ nanorods in Pd@Nb_2_O_5_. The Pd nanocrystals were found to be well dispersed on the surface of the Nb_2_O_5_ nanorods with diameter in the range of 2–10 nm.

It is well documented in the heterogeneous catalyst literature that specific surface area is important, where catalytic reaction sites and rates scale with surface area.^27^ The surface areas for the nanorod‐shaped Nb_3_O_7_(OH), Nb_2_O_5_ and Pd@Nb_2_O_5_ samples, determined using the Brunauer–Emmett–Teller (BET) method, were similar, namely 84.9, 82.1, and 82.4 m^2^ g^−1^, respectively.

**Figure 2:**
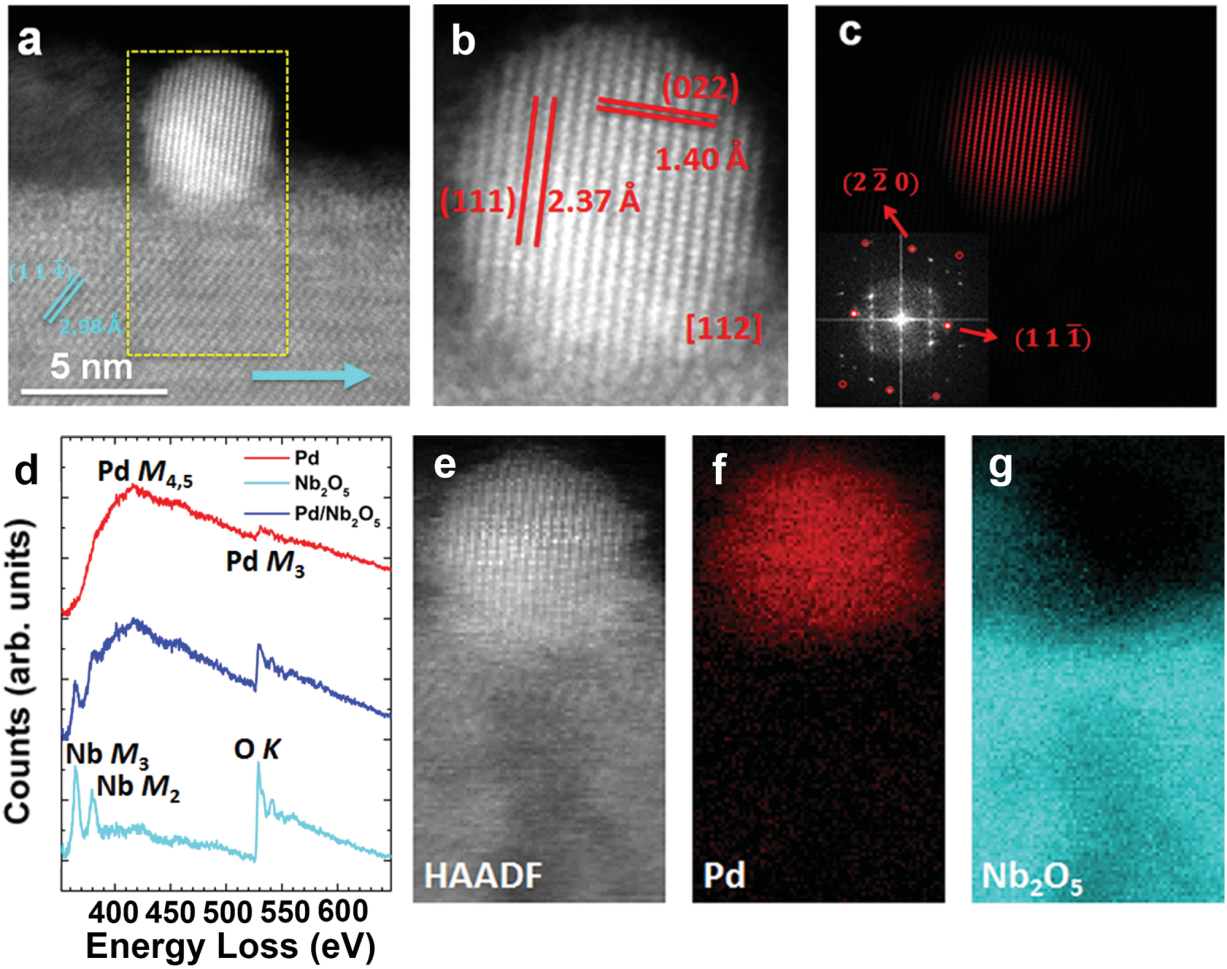
a) HAADF image of a Pd nanocrystal decorated Nb_2_O_5_ nanorod. The cyan arrow indicates the [010] growth direction of the nanorod, and the dashed yellow rectangle marks the area where EELS elemental mapping was taken. Scale bar, 5 nm. b) Enlarged image showing nanocrystalline Pd viewed from its [112] direction. The d‐spacing of the (111) and (022) planes was measured. c) Inverse FFT by masking spots only contributed from Pd, as indicated in the inserted figure by the red circles. The inserted figure is the FFT of (a), showing an overlap of contributions from Pd and Nb_2_O_5_. d) EEL spectra from the Pd nanocrystal (red), Nb_2_O_5_ nanorod (cyan), and interface between the Pd and Nb_2_O_5_ (blue). The Pd M_4,5_‐edge and Pd M_3_‐edge are visible in the red spectrum; the Nb M_3_‐edge, Nb M_2_‐edge and O *K*‐edge are visible in the cyan spectrum; a superposition of these edges are exhibited in the blue spectrum. e) HAADF image taken while simultaneously collecting the EELS map. f) Fitting coefficient of Pd showing the spatial distribution of the Pd signal. g) Fitting coefficient of Nb_2_O_5_ showing the spatial distribution of the Nb_2_O_5_ signal.

In order to determine the chemical state of the Pd nanocrystals, the samples were further analyzed by STEM and XPS. A HAADF Z‐contrast image of a Pd nanocrystal decorated Nb_2_O_5_ nanorod is shown in **Figure** **2**a. The atomic number difference provides a very clear image of a higher contrast Pd nanocrystal situated on top of a well‐defined Nb_2_O_5_ nanorod. The cyan arrow indicates the growth direction of the nanorod, and the lattice fringes corresponding to the $\left(1\;1\;\bar{4}\right)$ surface are visible in the nanorod. An enlarged image of the Pd nanocrystal, viewed down the [112] direction, is shown in Figure 2b. The d‐spacing values of the $\left(1\;1\;\bar{1}\right)$ and $(2\;\bar{2}\;0)0$ planes are 2.37 Å and 1.40 Å, respectively. Since the contrast of a HAADF image is not only determined by the Z number, but can also depend on thickness and channeling effects, we used the fast Fourier transform (FFT) to further verify the distribution of the Pd nanocrystals. The FFT of Figure 2a is shown in the inset of Figure 2c, where the spots from Pd and Nb_2_O_5_ nanostructures are visible, and the Bragg peaks from $\left(1\;1\;\bar{1}\right)$ and $\left(2\;\bar{2}\;0\right)$ planes are labeled. Figure 2c displays an inverse FFT, obtained by masking only the spots from Pd, as indicated by red circles in the insert. From the inverse FFTs we see that Pd nanocrystals only exist on the nanorod surface, and have a round shape.

We used electron energy‐loss spectroscopy (EELS) to further study the chemical composition of the Pd nanocrystal decorated Nb_2_O_5_ nanorods. Individual EEL spectra extracted from the nanocrystal, nanocrystal–nanorod interface and nanorod are plotted in Figure 2d. The spectra collected from the Pd nanocrystal shows the Pd M_4,5_‐edge and the Pd M_3_‐edge, while the spectra collected from the nanorod exhibits the Nb M_3_, Nb M_2_, and O K‐edges. As expected, the spectra collected at the interface between the Pd nanocrystal and the Nb_2_O_5_ nanorod show a superposition of these two individual spectra. Since the Pd M_4,5_‐edge overlaps with the Nb M_2_‐edge, and the Pd M_3_‐edge overlaps with the O K‐edge, we performed a multiple linear least square (MLLS) fitting analysis on the EELS mapping to distinguish those signals from each other. The spectra taken from Pd and Nb_2_O_5_ were used as references to obtain a fitting coefficient of Pd and Nb_2_O_5_. Figure 2e is a HAADF image taken simultaneously with the EELS mapping, as also marked in Figure 2a. The fitting coefficients of Pd and Nb_2_O_5_ are mapped in Figure 2f,g, respectively, showing the contribution of the corresponding reference spectra to the whole spectrum image. Again, the metallic Pd nanocrystal is found to be sitting on top of the Nb_2_O_5_ nanorod. Therefore, both atomic structure and elemental mapping validate the architecture of the Pd nanocrystal decoration on the Nb_2_O_5_ nanorod.

At the lowest Pd loading (0.5 wt%), we were not able to determine the chemical state of Pd by performing XPS measurements. However, by using a Pd@Nb_2_O_5_ sample with double the Pd loading (1 wt%), the Pd XPS signal could be discerned where binding energies of Pd 3d at 335.6 eV and 340.8 eV are assigned to the 3d_5/2_ and 3d_3/2_ spin‐orbit peaks of metallic Pd, further validating the accumulating evidence that Pd nanocrystals are in the zero‐valent metallic state in the Pd@Nb_2_O_5_ sample (Figure S4, Supporting Information).^28^

**Figure 3:**
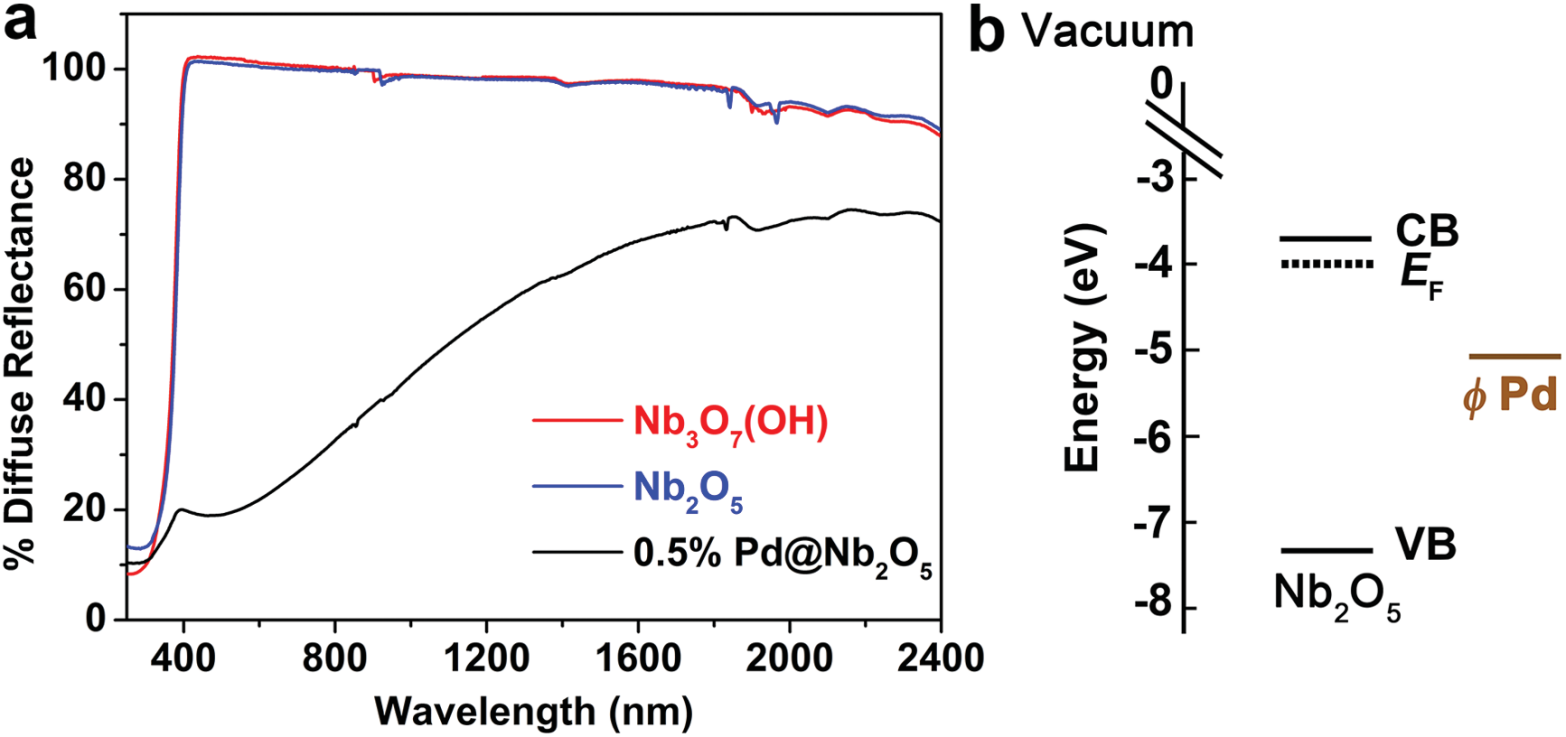
a) Diffuse reflectance spectra of Nb_3_O_7_(OH), Nb_2_O_5_ and Pd@Nb_2_O_5_ films dispersed on a borosilicate filter. b) The band energy diagram of Nb_2_O_5_ in comparison with the work function of Pd.

The optical properties of different samples will now be presented. **Figure** **3**a depicts the diffuse reflectance spectra of each sample measured over the spectral range from 250 to 2400 nm. The diffuse reflectance of the Pd@Nb_2_O_5_ sample is much less than that of the Nb_3_O_7_ (OH) and Nb_2_O_5_ samples throughout the visible and NIR spectral regions because a significant amount of light is absorbed by the Pd nanocrystals. In the literature, the difference between insulating wide bandgap catalyst supports with and without Pd loaded onto their surface has been attributed to light absorption by the Pd nanoparticles.^10^, ^11^ The optical bandgap of Nb_2_O_5_ was determined to be 3.44 eV by fitting the diffuse reflectance spectra with a modified Kubelka–Munk function, as shown in Figure S5 (Supporting Information). From our XPS results (Figure S6, Supporting Information), the valence band maximum (VB) and Fermi energy (E*_f_*) of the Nb_2_O_5_ nanorods were determined to be 7.31 eV and 4.01 eV, respectively. The work function of Pd is reported in the literature.^29^ By combining the optical band gap with the data obtained from XPS (Figure S6, Supporting Information), the band energy diagram of Nb_2_O_5_ can be constructed (Figure 3b).

According to the literature,^15^ based on Mie theory, the plasmon resonance of Pd nanocrystals smaller than 10 nm in diameter locates in the UV wavelength region. Due to the overlapping of the bandgap absorption of the Nb_2_O_5_ nanorods with the plasmon resonance of the small Pd nanocrystals, no peak associated with the SPR of Pd in the UV range was observed in Figure 3a. It is well known that the contributions from the SPR effect and interband and intraband transitions vary from metal to metal.^10^ For these small Pd nanocrystals, the optical absorption in the visible and NIR spectral range (400–2400 nm) have been well documented and mainly attributed to the interband and intraband transitions.^10^, ^11^, ^14^, ^16^

### 2.2 Investigation of Photothermal RWGS Catalysis on Nanostructured Pd@Nb_2_O_5_

In order to determine the photothermal catalytic activity of nanostructured Pd@Nb_2_O_5_ towards the RWGS, ≈4 mg samples were deposited onto a borosilicate filter support having an area of ≈2.2 cm^2^. The sample was loaded into a custom built stainless steel batch reactor with a volume of 1.8 mL. The reactor was loaded with CO_2_ and H_2_ gas at 1:1 ratio to a total pressure of 27 psi. Three tests were then performed on this catalyst sample; (1) in the dark at room temperature (RT), (2) under irradiation from a 300 W Xe lamp at 25 kW m^−2^, and (3) in the dark at a temperature of 160 °C. Moreover, in this work all reported rates are normalized to the weight of Pd since the active sites reside at the Pd@Nb_2_O_5_ interface, as will be described further in Section 2.5.

**Figure 4:**
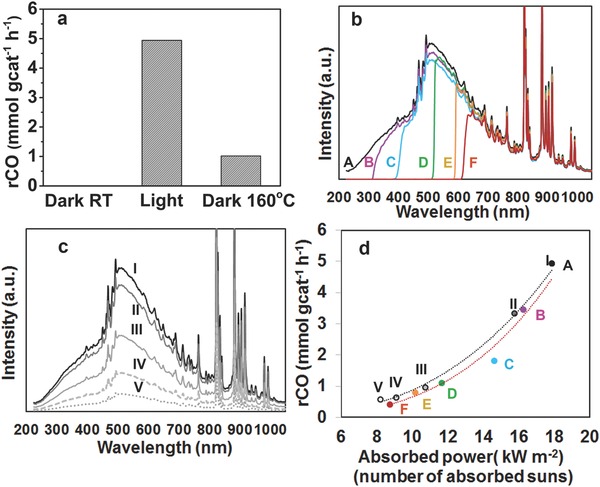
Photothermal catalytic performance of the nanostructured Pd@Nb_2_O_5_ samples. a) CO production rates over Pd@Nb_2_O_5_ in the dark at room temperature (RT), under irradiation from a 300 W Xe lamp, and in the dark at a reaction temperature of 160 °C. b) Spectral irradiance incident onto the Pd@Nb_2_O_5_ catalyst with different cut‐off filters for batch reaction tests A through F. c) Spectral irradiance incident onto the Pd@Nb_2_O_5_ catalyst for batch reactions I through V. d) The RWGS reaction rates plotted as a function of absorbed power for the series of batch reactions A–F (red line) and *I*–*V* (black line).

The duration of each test was 3 h and the results are plotted in **Figure** **4**a. Furthermore, isotope tracing experiments using carbon‐13 labeled carbon dioxide were performed in order to confirm with complete certainty that the ^13^CO products originated from ^13^CO_2_ rather than adventitious carbon sources (see Figure S7 in the Supporting Information). The results in Figure 4a show that the RWGS reaction does not occur in the dark at room temperature, while under irradiation from the 300 W Xe lamp at an incident intensity of 25 kW m^−2^, we observe a CO_2_—to—CO hydrogenation rate as high as 4.9 mmol g_cat_^−1^ h^−1^ over the hybrid Pd@Nb_2_O_5._ For comparison, the reaction proceeded at a rate of 1 mmol g_cat_^−1^ h^−1^in the dark at 160 °C. The catalytic rate in the light is roughly five times the rate in the dark under thermal heating, demonstrating that the RWGS reaction can be efficiently driven solely by light over the nanostructured Pd@Nb_2_O_5_ catalyst. It can also be noted that no ^13^C labeled CH_4_ product was present for either the photo‐ or thermal catalytic reaction tests, with the exception of some tests carried out under the highest light intensity. That is, for some tests wherein the intensity of the incident light was 25 kW m^−2^, small amounts of ^13^CH_4_ product were visible. However, it should be noted that even for these cases the CH_4_ production rate was typically less than two orders of magnitude of the ^13^CO production rate and never exceeded 5% of the CO production rate for any given test.

### 2.3 Investigating the Effect of Spectral Distribution and Light Intensity on the Photothermal Catalytic Activity

In order to quantitatively investigate the effects of the spectral power distribution on the CO production rate, a series of tests was carried out wherein the Pd@Nb_2_O_5_ catalyst sample was irradiated with a 300 W Xe lamp, fitted with different high‐pass cut‐off filters, and the sample was exposed to air for 24 h between each test. The relative spectral distribution for this series of tests, labeled A through F, is shown in Figure 4b such that for A: no filter, B: λ > 295 nm, C: λ > 380 nm, D: λ > 495 nm, E: λ > 590 nm, and F: λ > 615 nm. For test A, the CO hydrogenation reaction proceeded at a rate of 4.9 mmol g^−1^ h^−1^. The CO production rates for batch reactions A through F are plotted in Figure 4d. It is noteworthy that the RWGS rates for reaction tests C, D, E, and F are 1.8 mmol g_cat_^−1^ h^−1^, 1.1 mmol g_cat_^−1^ h^−1^, 0.78 mmol g_cat_^−1^ h^−1^ and 0.41 mmol g_cat_^−1^ h^−1^, respectively. For test C, the CO_2_ hydrogenation reaction proceeded at a rate of 1.8 mmol g_cat_^‐1^ h^−1^ using photons in the visible and NIR region. This suggests the RWGS reaction can be driven by visible and NIR light over nanostructured Pd@Nb_2_O_5_ catalysts.

We also investigated the light intensity dependence of the CO production rates. Specifically, a series of tests labeled I through V was carried out wherein the intensity of the incident light from the 300 W Xe Lamp was sequentially reduced, and the sample was exposed to air for 24 h between different tests. No filters were used and the relative spectral irradiance for tests I through V are plotted in Figure 4c. The RWGS reaction rates for the series of tests labeled I through V are plotted as a function of absorbed irradiated power in Figure 4d alongside the reaction rates for tests A through F. The absorbed radiant power was determined by multiplying the spectral irradiance with the absorption spectra, which was determined using the equation *A* = *1* − *T* − *R*, where *R* and *T* are the diffuse reflectance and transmittance, respectively.

The RWGS reaction rates for the series of tests A through F and I through V are plotted as the red and black dashed lines in Figure 4d, respectively. The fact that these red and black curves nearly overlap indicates that the RWGS reaction rates primarily depend on the total amount of absorbed radiant energy rather than the spectral power distribution of the radiant energy. For example, consider tests III and E wherein the amount of absorbed radiant energy are very close at values of 10.8 kW m^−2^ and 10.2 kW m^−2^, respectively. The RWGS rates for these two tests are also nearly identical despite the fact that the nanostructured Pd@Nb_2_O_5_ catalyst is primarily irradiated with NIR photons (*λ* > 600 nm) for test E whereas for test III more than 50% of the incident radiant energy is from the spectral region with *λ* < 600 nm. Here, it should also be noted that photons with energy greater than the bandgap of Nb_2_O_5_ (*λ* < 360 nm) account for more than 5% of the incident radiant energy in test III whereas no photons in this spectral region are incident on the Pd@Nb_2_O_5_ catalyst in test E. These results imply that electron–hole pairs photogenerated in the Nb_2_O_5_ nanorods (*E*_g_ = 3.44 eV) upon absorption of UV photons or ultraviolet plasmonic excitation of the Pd nanocrystals are not the primary factors driving the RWGS reaction. Thus, we can make the following two statements based on the results shown in Figure 4:

1) Considering the visible and NIR spectral range, where the energy of incident photons is less than the bandgap of Nb_2_O_5_ and less than the plasmon resonance energy of Pd nanocrystals, one can deduce that photons absorbed in the nanostructured Pd@Nb_2_O_5_ catalyst provide thermal energy caused by intraband and/or interband electronic transitions and nonradiative relaxation. This activates the RWGS reaction photothermally.
2) Considering the UV spectral range, photons have sufficient energy to activate the RWGS photochemically by generating electron–hole pairs in the Nb_2_O_5_ support or by exciting local plasmon resonance in Pd nanocrystals. While these photochemical reaction pathways contribute to the overal reaction rates, they are not the main driving force for the RWGS reaction over the nanostructured Pd@Nb_2_O_5_ catalysts. If the photochemical contribution was the main driving force then the dashed black line corresponding to tests I through V in Figure 4d would be significantly higher than the dashed red line.

Therefore, we propose that, under illumination, absorbed photons ultimately thermalize and generate heat, resulting in high local temperatures in the Pd@Nb_2_O_5_ catalyst, which photothermally accelerates the RWGS reaction.

A TEM image of the sample taken after these photothermal catalytic tests is shown in Figure S8 (Supporting Information), notably illustrating that there is no observable change in nanostructure morphology.

### 2.4 Investigating the Effect of the Temperature Dependence of RWGS Reaction Rates over Pd@Nb_2_O_5_ in the Dark

Since it is clear that the RWGS is driven photothermally, we were interested in determining the temperature of the nanostructured Pd@Nb_2_O_5_ catalyst as a function of the intensity and spectral distribution of the incident radiant power.

One approach was to determine an “effective” reaction temperature of the catalyst, *T*_e_, defined herein as the catalyst temperature associated with a given reaction rate attained when the catalyst was tested under similar reaction conditions but in the dark. Therefore, in order to estimate *T*_e_ for the nanostructured Pd@Nb_2_O_5_ catalyst during tests at RT when subjected to incident radiant energy, the film sample was tested at temperatures of 60 °C, 85 °C, 110 °C, 135 °C, and 160 °C in the dark.

**Figure 5:**
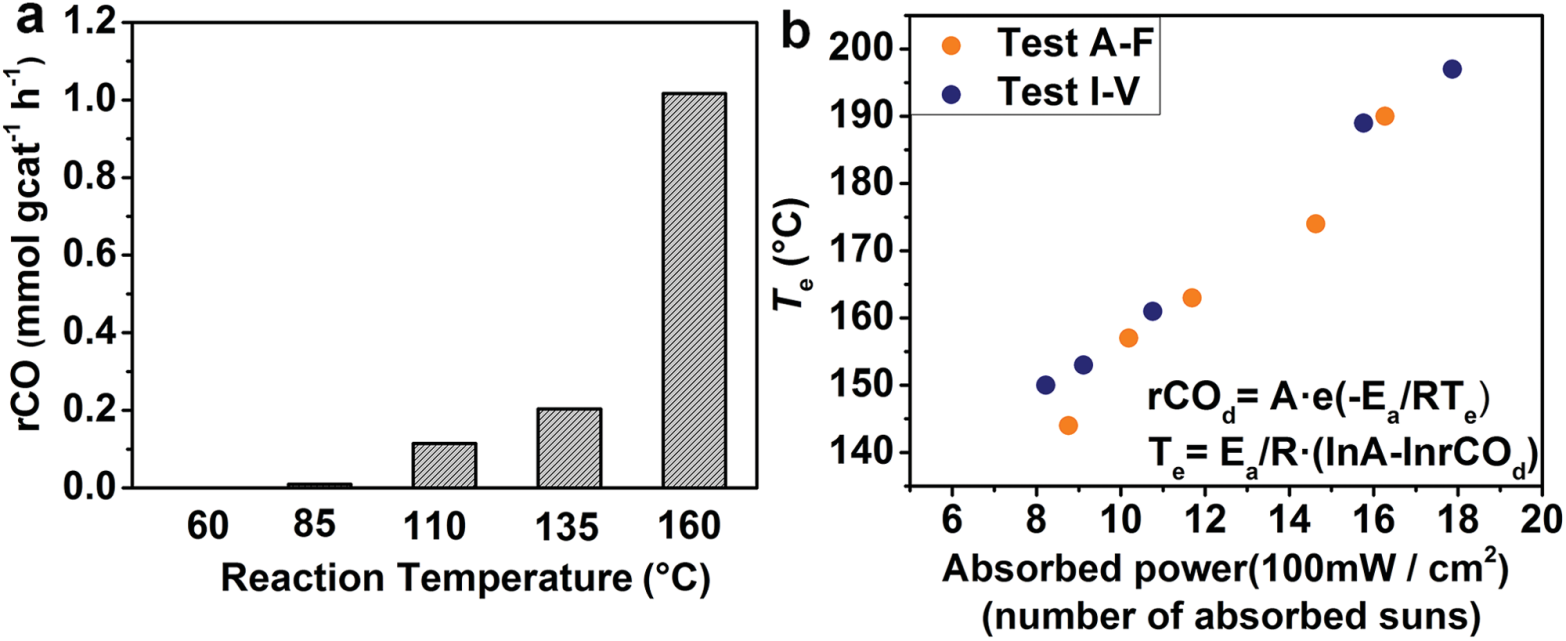
a) RWGS reaction rate measured in the dark as a function of reaction temperature. b) The effective reaction temperature as a function of radiant power absorbed by the Pd@Nb_2_O_5_ catalyst. The equation used to estimate *T*_e_ is provided in Figure b.

We fit the reaction rates measured for these dark tests, which are plotted in **Figure** **5**a, to an Arrhenius rate equation in order to determine an effective reaction temperature as a function of the CO production rate. As shown in Figure 5b and Table S1 (Supporting Information), the effective temperature of the catalyst increases from about 140 °C to 200 °C as the absorbed radiant power increases from ≈8 kW m^−2^ to 18 kW m^−2^.

### 2.5 Investigating the RWGS Reaction Pathway over Pd@Nb_2_O_5_

**Figure 6:**
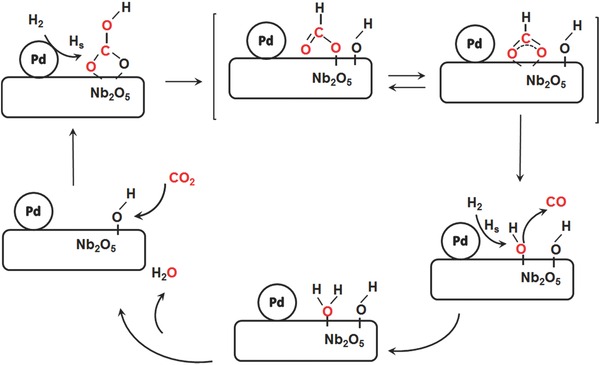
Possible reaction pathway for the reverse water gas shift reaction (RWGS) on the Pd@Nb_2_O_5_ hybrid catalyst.

Our justification for using an Arrhenius rate law in the previous subsection is based on previous studies that report a kinetic model for the thermally driven catalytic conversion of CO_2_‐to‐CO over M@M'O*_x_* catalysts.^22^, ^24^, ^30^, ^31^ The widely accepted pathway is the formate conversion mechanism, as shown in **Figure** **6**. Atomic hydrogen species are formed via chemisorption and dissociation of H_2_ on M, which then migrate (spillover) to the M‐M'O*_x_* interface and diffuse onto the M'O*_x_*, acting as a reducing species. Formate is formed in the CO_2_ chemisorption and hydrogenation process on the surface of the M'O*_x_* support, followed by a rearrangement that generates CO and surface OH.

In order to investigate if the RWGS proceeds by means of the formate decomposition pathway shown in Figure 6 over the Pd@Nb_2_O_5_, we first conducted controlled catalytic measurements over Pd@Nb_2_O_5_ under illumination in the presence and absence of H_2_, as shown in Figure S9 (Supporting Information). The results confirm that the reduction of CO_2_ can happen only in the presence of H_2_.

We investigated the CO_2_ adsorption capacity of the Pd@Nb_2_O_5_ catalysts using thermogravimetric analysis. The CO_2_ adsorption capacities are normalized to the surface area of each sample, which was measured using the BET method. The CO_2_ adsorption capacities are 1.11 μmol CO_2_ m^−2^ and 1.13 μmol CO_2_ m^−2^ for Nb_2_O_5_ and Pd@Nb_2_O_5_, respectively. These values are very similar. The similarity in CO_2_ adsorption for the Nb_2_O_5_ and Pd@Nb_2_O_5_ samples suggests that Pd does not significantly affect the availability of surface‐bound CO_2_ species.

We also considered the reaction rate activation energy. In this context it has been shown that the most likely rate‐limiting step for CO formation involves the conversion of formate intermediates to CO at the metal/oxide interface according to the reaction scheme:

$${\mathrm{HCO}}_{2}^{-*}↔{\mathrm{HO}}^{-*}+\mathrm{CO}\left(g\right)$$

The activation energy of this rate‐limiting step is reported to be 66 kJ mol^−1^.^22^ In comparison, using the Arrhenius rate law equation, we determine for nanostructured Pd@Nb_2_O_5_ an activation energy of 76 kJ mol^−1^. Indeed, the fact that these activation energies are comparable provides further evidence that the RWGS likely proceeds over the Pd@Nb_2_O_5_ catalyst via the formate decomposition pathway shown in Figure 6.

To clarify whether or not any carbon was deposited on the surface of the Pd@Nb_2_O_5_ catalyst during the reaction, we performed both hydrogenation and thermogravimetric analysis (TGA) measurements on fresh and used catalysts. To conduct the hydrogenation experiment over used Pd@Nb_2_O_5_ catalysts that had been tested under the high light intensity testing conditions for over 40 h, we infiltrated the photoreactor with pure hydrogen gas at a pressure of 2 atmospheres for a duration of 3 h under an intensity of 25 kW m^−2^. No carbonaceous species were detected in the hydrogen gas after the 3 h test. We then repeated this hydrogenation test with the reactor heated to a temperature of 150 °C, and were still unable to detect any carbonaceous species. The TGA experiments were performed on Pd@Nb_2_O_5_ catalysts that had been used under high light intensity testing conditions reported in the paper for over 40 h.^32^ The results from the TGA analysis, plotted in Figure S10 (Supporting Information), showed no weight loss in the temperature range of 400–700 °C for two tests, one performed under flowing CO_2_ and the other flowing air. The TGA results for the used catalyst are identical to those for a fresh catalyst. Thus, the results from both our hydrogenation and TGA experiments imply that the surface of the Pd@Nb_2_O_5_ catalyst is devoid of surface carbon after catalyzing the photothermal reduction of CO_2_.

### 2.6 Raman Investigation of the Visible Light Induced Photothermal Effect

**Figure 7:**
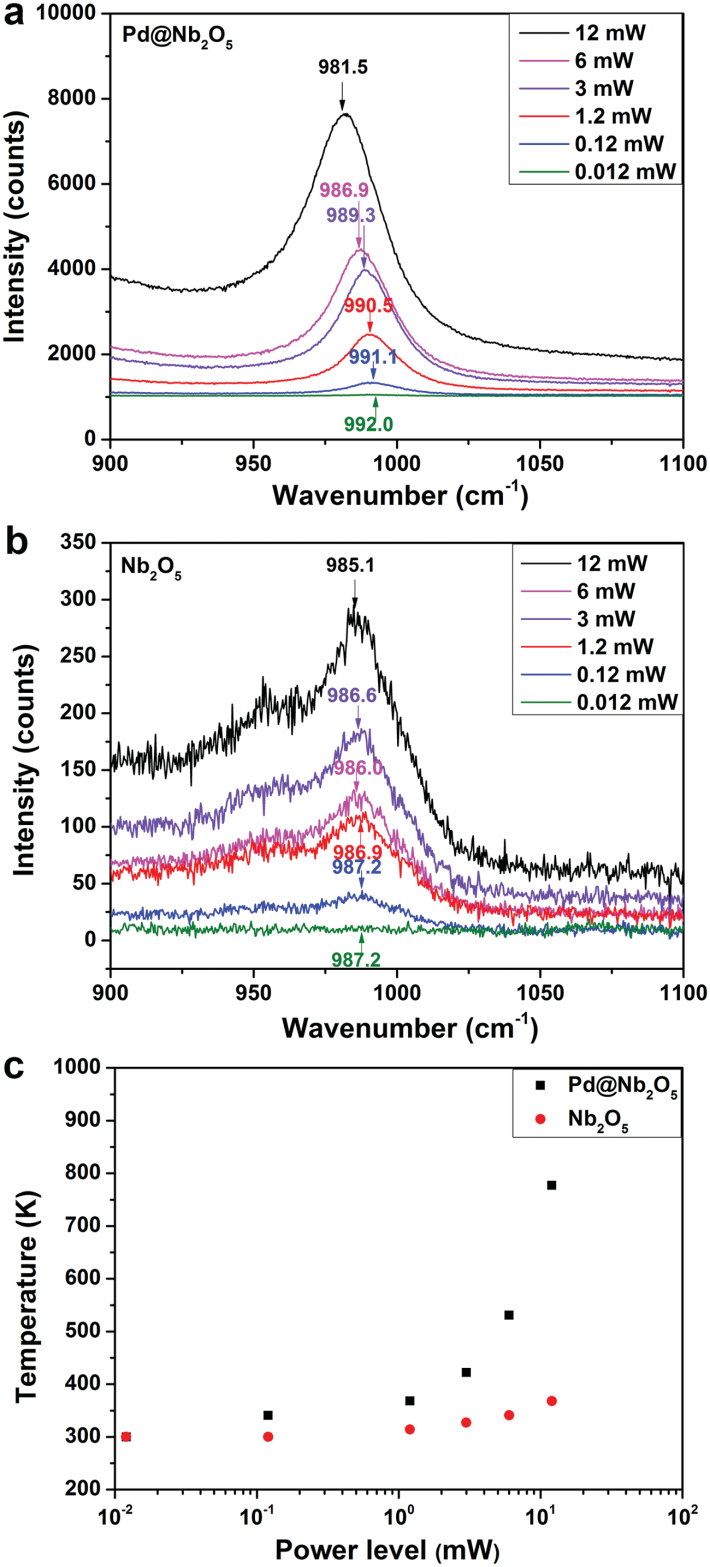
Dependence of the Raman frequency for νNb=O stretching vibrations of a) Pd@Nb_2_O_5_ and b) Nb_2_O_5_ nanorods at different power levels. c) Estimated temperatures for Pd@Nb_2_O_5_ and Nb_2_O_5_ at different power levels.

It is important to examine the local temperature of the nanostructure surface under irradiation in order to understand the underlying mechanisms associated with the photothermal effect and determine the specific roles played by the metal nanocrystals and supports. To further understand the impact that the photothermal effect has on the temperature of the sample, Raman measurements were conducted on nanostructured Pd@Nb_2_O_5_ and Nb_2_O_5_ in order to more directly measure the local temperature that the samples attain in the light. In 2001, Bell conducted a pivotal investigation of the effects of temperature on the intensities and positions of Raman bands of metal oxides.^33^ It was found that, with increasing temperature, all Raman bands of metal oxides shifted to lower frequencies. This was attributed to thermal expansion of the lattice and changes in the population of the vibrational energy levels with increasing temperature. For Nb_2_O_5_, a linear shift in the position of the band for niobium‐oxygen vibrations was observed as the acquisition temperature increased.^33^ In our work, we utilized a 633 nm laser to record Raman spectra for Pd@Nb_2_O_5_ and Nb_2_O_5_ at 6 power levels, as shown in Figure S11 (Supporting Information). The νNb=O stretching modes for Pd@Nb_2_O_5_ and Nb_2_O_5_ at around 990 cm^−1^ under different power levels are shown in **Figure** **7**. It is important to note that under higher power levels, the band position for the νNb=O stretching mode of Pd@Nb_2_O_5_ exhibited a shift to low frequencies. Under the lowest power level where useful Raman spectra are attainable, which is found to be 12 μW (laser power density is 2.4 kW cm^−2^), we assume that the temperatures of Pd@Nb_2_O_5_ and Nb_2_O_5_ are both approximately 300 K. This assumption is based on recent work by Kim et al. wherein Raman measurements performed with laser excitation at a wavelength of 532 nm and power density of 0.670 kW cm^−2^ on Bi_2_Se_3_ and Sb_2_Te_3_ crystals did not increase their temperature above 300 K.^34^ Given that the power density reported by Kim et al. is comparable to that used in our experiments, and also given the larger energy of the pump used in their work (532 nm) in comparison to the energy of the pump photons used in our work (633 nm) in relation to the small bandgap of Bi_2_Se_3_ and Sb_2_Te_3_ (≈0.3 eV), we do not expect the temperature of our Pd@Nb_2_O_5_ and Nb_2_O_5_ to be significantly elevated above room temperature when subjected to laser excitation at the lowest laser power density of 2.4 kW cm^−2^.

We estimated the temperatures of Pd@Nb_2_O_5_ and Nb_2_O_5_ at the other 5 power levels using the linear dependence of the band shift with temperature based on Bell's findings, as shown in Figure 7c and Table S2 (Supporting Information). Notably, the temperature of Pd@Nb_2_O_5_ at the highest laser power level of 12 mW (laser power density is 2388 kW cm^−2^) is estimated to be 777 K while the temperature of Nb_2_O_5_ at the same power level is estimated to be 368 K.

In addition to the aforementioned approach involving temperature‐dependent Raman mode frequency shifts, one can directly and quantitatively determine the local temperature of the Pd@Nb_2_O_5_ and Nb_2_O_5_ sample using the correlation between the intensities of the Stokes and anti‐Stokes components of the Raman spectra where the local temperature of the sample can be determined from the expression:^35^

(1) $$\frac{I_{\mathrm{ASt}}}{I_{\mathrm{St}}}={\left(\frac{\omega_{L}+\omega_{V}}{\omega_{L}-\omega_{V}}\right)}^{4}e^{-E_{R}/\left(kT\right)}$$

Here the intensities of the signals, *I*_St_ and *I*_ASt_, are the integral of the collected Raman signals from the Stokes (S) and anti‐Stokes (AS) Raman modes, respectively; T is the temperature in degrees Kelvin; *E*_R_ = *hc*/*λ*_excitation_ − *hc*/*λ*_emission_ = *hc ω*_V_, where *ω*_V_ is the temperature‐dependent wave number of the Raman mode (cm^−1^); *ω*_L_ is the wave number of the exciting laser light (12739 cm^−1^), giving ((*ω*_L_ + *ω*_V_)/(*ω*_L_ – *ω*_V_))^4^ = 1.492.

In order to determine the local temperature of the sample, both the Stokes and weaker anti‐Stokes Raman spectra of Pd@Nb_2_O_5_ and Nb_2_O_5_ were measured under a similar power density of 3050 kW cm^−2^ (24 mW) using a pump wavelength of 785 nm. The Raman signals of the Stokes and anti‐Stokes bands for the νNb—O—Nb stretching mode around 640 cm^−1^ are plotted in Figure S12 (Supporting Information).^36^ The obtained values of the Raman signals of the Stokes modes, anti‐Stokes modes, the ratio of Stokes to anti‐Stokes peaks, and the estimation of the temperatures are shown in Table S3 (Supporting Information). The temperatures of Pd@Nb_2_O_5_ and Nb_2_O_5_ are estimated to be 776 K and 385 K, respectively.

The local temperatures of Pd@Nb_2_O_5_ and Nb_2_O_5_ determined by the two methods are consistent and clearly demonstrate that there is a photothermal effect of the Pd nanocrystals decorated on the Nb_2_O_5_ nanorods. The increase in the temperature of the Nb_2_O_5_ nanorods alone at different laser power levels is minimal, while that of Nb_2_O_5_ nanorods decorated with the Pd “nano‐heaters” can be increased from 300 K to around 777 K by increasing the pump laser power densities. This can be attributed to the photothermal effect of the Pd nanocrystals. Since we were using 633 nm and 785 nm laser light, it is also confirmed that the photothermal effect of the Pd nanocrystals can be induced by visible irradiation. It is important to note that the Raman measurements presented in this work demonstrate the photothermal effect on the Pd@Nb_2_O_5_ catalyst and also provide valuable insight directly demonstrating how the Pd nanocrystals heat up to a much greater extent than the Nb_2_O_5_ nanorod supports. However, it is equally important to point out the distinction between the laser excitation used in the Raman measurement when compared to broadband solar irradiation. Both have different brightness and associated spectral width, and as such, the measurements reported here are not used to determine exact estimates of the temperature of the Pd@Nb_2_O_5_ catalyst during the reaction in the light because the laser light source is monochromatic compared to the polychromatic output of the Xe lamp used to measure the RWGS reaction rates. Nevertheless, the Raman experiments performed herein allow us to infer that the comparative photothermal heating and temperature increase of the Pd@Nb_2_O_5_ nanocrystal–nanorod can be attributed to the interband and intraband absorption contributed by the Pd nanocrystals. The measurements provide direct evidence that this optical absorption is the underlying physicochemical phenomenon driving the RWGS reaction over the nanostructured Pd@Nb_2_O_5_.

### 2.7 Investigating In Situ Generated Oxygen Vacancies and Reduced Oxidation State Surface Niobium Sites in the Photothermal Catalytic CO_2_ Reduction of Nanostructured Pd@Nb_2_O_5_

**Figure 8:**
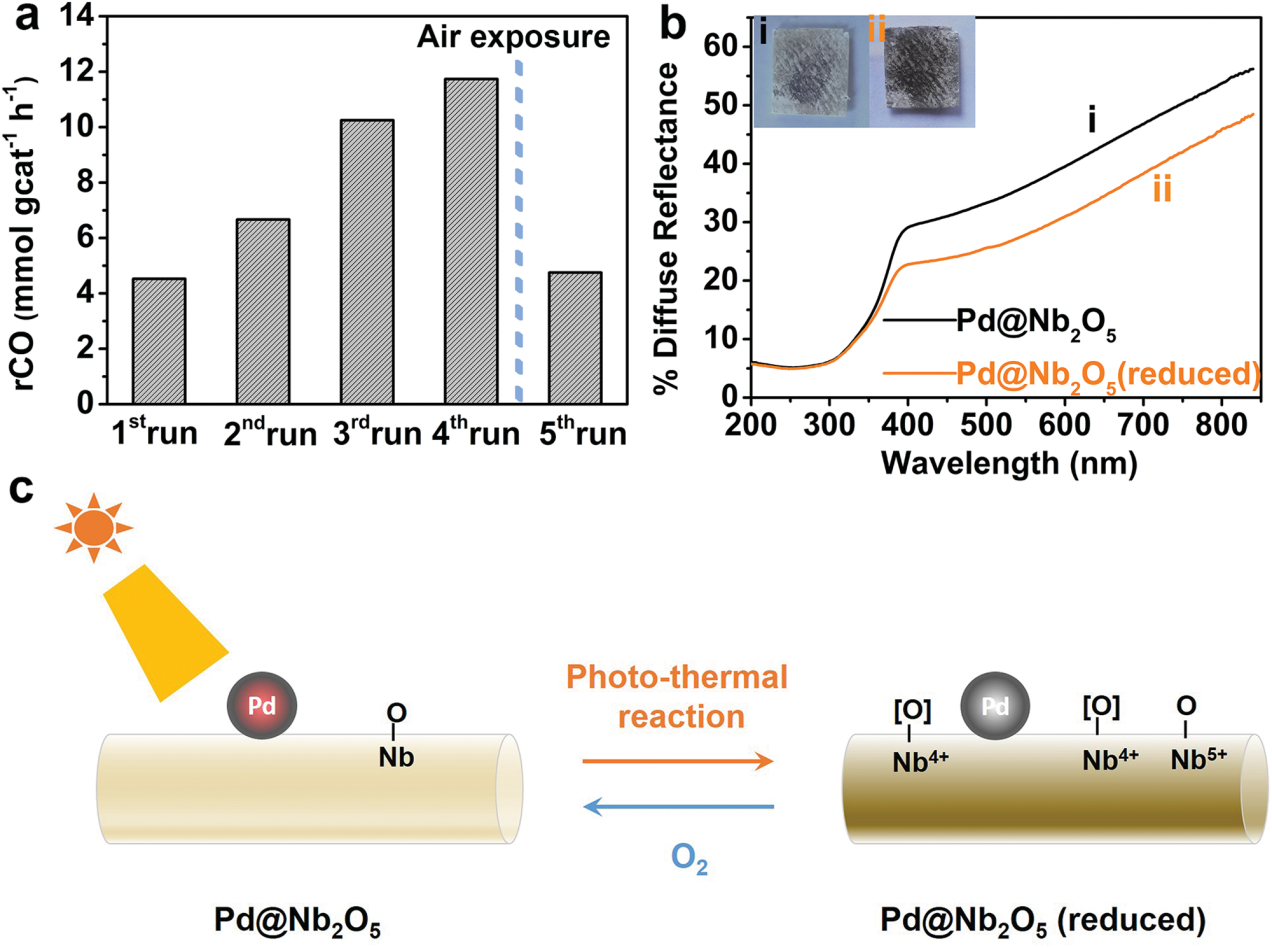
Photothermal catalysis by nanostructured Pd@Nb_2_O_5_ (reduced) sample. a) Without exposure to air, the production rates of CO over Pd@Nb_2_O_5_ kept monotonically increasing from the first run to the fourth run. When the sample was exposed to air for 24 h, the production rate of the fifth run dropped to that of the first. b) Diffuse reflectance spectra and appearance of (i) pristine Pd@Nb_2_O_5_ and (ii) reduced Pd@Nb_2_O_5_ nanostructured films. c) Schematic illustration of in situ generation of reduced surface niobium sites and/or oxygen vacancies during photothermal reaction.

Often, the activity of photocatalysts decrease gradually with sequential tests due to numerous possible reasons including sintering, photodegradation, catalyst poisoning, and adventitious contamination. However, in contrast, our photothermal catalysts display a gradual rate enhancement with the time of testing. To gain a deeper insight into this unusual behavior, a set of photothermal catalytic measurements was carried out under similar reaction conditions as the tests reported in Figure 4d with the sample kept under vacuum between different measurements. In **Figure** **8**a, it can be seen that when the Pd@Nb_2_O_5_ sample was initially irradiated, a CO production rate of 4.5 mmol g_cat_^−1^ h^−1^ was observed for a 3 h test. The second run produced CO at a rate of 6.7 mmol g_cat_^−1^ h^−1^ and the third run produced CO at a rate of 10.2 mmol g_cat_^−1^ h^−1^. For the fourth run, the RWGS proceeded at a rate of 11.7 mmol g_cat_^−1^ h^−1^, which is 2.6 times that of the first run. Finally, the film sample was exposed to air for 24 h and a repeat measurement was conducted, more or less reproducing the original rate of 4.7 mmol g_cat_^−1^ h^−1^. These results demonstrate that a certain kind of chemical/surface species were generated in situ during the catalyst testing process and these species, which exist under vacuum or H_2_ and CO_2_ gas atmosphere, are influencing the photothermal catalytic reaction in a positive way. Typically, on metal oxide supports such as TiO_2_, CeO_2,_ and Nb_2_O_5,_ oxygen vacancies (Vo) and reduced surface states (Ti^3+^, Ce^3+^ and Nb^4+^) can be formed by annealing in an inert environment at high temperatures or under illumination.^18^, ^30^, ^37^, ^38^, ^39^, ^40^ In this context, oxygen vacancies and reduced surface states can increase the affinity of CO_2_ to the surface, lifetime of photogenerated charge carriers and visible light absorption.

Figure 8b shows the diffuse reflectance spectra and appearance of the Pd@Nb_2_O_5_ film sample before and after conducting the photothermal catalytic tests. When oxygen vacancies are generated in metal oxides, they can create mid‐gap color centers, enhancing the harvesting of visible light.^18^, ^37^, ^41^ Reduced surface states can also be created in metal oxides, generating sub‐bandgap states below the conduction band and causing the metal oxide to absorb more visible light due to inter‐valence‐charge‐transfer or plasmonic effects.^19^ It was found that the color of the film sample became observably brownish and the visible light absorption increased, indicating that oxygen vacancies and/or surface reduced niobium oxidation states are formed after light irradiation during the photothermal catalytic measurements. To confirm that oxygen vacancies and corresponding reduced niobium oxidation states (Nb^4+^) are generated under irradiation conditions, low temperature EPR spectra were recorded on Nb_2_O_5_ nanorod samples (Figure S13, Supporting Information). Without any illumination, no EPR signal associated with Nb^4+^ could be observed.^39^ After the Nb_2_O_5_ nanorod sample had been illuminated under a 300 W Xe lamp, an EPR signal with *g* = 1.9 was detected, which is diagnostic of Nb^4+^. This confirms that Nb^4+^ species can be formed through the reduction of Nb^5+^ under irradiation.

It has been demonstrated in the literature that oxygen vacancies and reduced oxidation state surface metal sites can increase the proclivity towards the adsorption and dissociation of CO_2_ on the surface of the metal oxide support, thereby promoting the formation of reaction intermediates and products.^30^, ^42^ Intermediates could be reduced to CO via reaction with spillover hydrogen and also by direct dissociation while healing the Vo sites.^18^, ^37^ To investigate these potential reaction pathways further, we conducted controlled catalytic measurements over reduced Pd@Nb_2_O_5_ under illumination. Firstly, for 3h tests with just CO_2_ at a reaction pressure of 27 psi, in the absence of H_2_, no CO was detected. Furthermore, 3 h tests with CO_2_ and H_2_ at 27 psi and a 1:1 gas ratio carried out in the dark did not yield any CO product. Based on the reaction rate measurements presented in Figure 4 and Figure 8 and the results from the aforementioned control experiments, we propose the following reaction mechanism. Under irradiation, H_2_ is dissociated on the photothermally heated Pd nanocrystals and so‐formed H migrates to the surface of Nb_2_O_5_. Concomitantly, Pd nanocrystals behave as local nanoscale heat sources enabling the photothermal effect on Nb_2_O_5_ nanorods to occur, where adsorbed CO_2_ and H_2_ based surface intermediates transform to CO and H_2_O. When oxygen vacancies and/or surface reduced niobium states are formed, the affinity of the surface of Nb_2_O_5_ for CO_2_ is increased. Moreover, the existence of oxygen vacancies and reduced niobium oxide surface states favour the decomposition of intermediates and are found to increase the CO production rate by a factor of 2.6.^18^, ^30^, ^37^, ^41^

## 3 Conclusion

In this study, the efficient photothermal catalytic activity of Nb_2_O_5_ nanorod‐supported Pd nanocrystals towards the reduction of CO_2_ to CO in the presence of H_2_ has been demonstrated. By powering this photothermal reaction with visible and near‐infrared light, conversion rates as high as 1.8 mmol g_cat_^−1^ h^−1^ have been achieved.

By comparing the intensity and spectral power dependence of the RWGS conversion rate, it was found that the photothermal catalytic effect originates from intraband and/or interband optical excitation and nonradiative relaxation of Pd nanocrystals rather than being driven by ultraviolet plasmon excitation of Pd nanocrystals or electron–hole pair generation upon absorption of UV photons in the Nb_2_O_5_ support.

The Raman measurements presented in this study demonstrate the photothermal effect of the Pd “nanoheaters” on the Nb_2_O_5_ nanorod supports and also provide a valuable probe of the local temperature. The results of this study also suggest that the observed RWGS reaction rate enhancement by a factor of 2.6 on nanostructured Pd@Nb_2_O_5_ that occurs during catalytic measurements is associated with in situ generation of oxygen vacancies and/or reduced niobium oxidation states on the surface of the Nb_2_O_5_ nanorods, which provides an additional driving force to promote the adsorption and reaction of CO_2_ with H_2_ to form CO and H_2_O.

It is hoped that the knowledge gleaned from this investigation will contribute to the design, synthesis and optimization of highly efficient photothermal catalysts that use the full solar spectrum to convert gas‐phase CO_2_ to valuable chemicals and fuels.^43^

## Experimental Section

*Synthesis of Nb_3_O_7_(OH) Nanorods*: Niobium powder (680 mg of Nb, 325 Mesh, Aldrich) was dissolved in a hydrochloric acid solution (9 mL HCl, Sigma–Aldrich; 10 mL deionized water) in a pyrex beaker. The aqueous solution was ultrasonicated for half an hour and then stirred for 15 min. The solution was subsequently placed in a Teflon‐lined stainless steel autoclave with 100 mL capacity. The hydrothermal reaction was performed at *T* = 200 °C for 24 h.^40^ After cooling to room temperature, the white product was collected through a centrifugation process and washed three times with deionized water to remove non‐reacted residues. Finally, the sample was dried in a vacuum oven at *T* = 70 °C for 12 h.

*Synthesis of Nanostructured Pd@Nb_2_O_5_*: Nanostructured Pd@Nb_2_O_5_ was synthesized via a microwave‐assisted reaction. Typically, 50 mg of Nb_3_O_7_(OH) nanorods were suspended in anhydrous ethanol (20 mL) in a pyrex vessel with 40 mL capacity. A stock solution of Na_2_PdCl_4_ (39.3 mg, Na_2_PdCl_4_·3H_2_O, Alfa Aesar) was prepared in anhydrous ethanol (20 mL), the concentration of which is 1 mg mL^−1^. 0.25 mL of Pd precursor solution, equivalent to 0.5 wt%, was added to the dispersion of 50 mg Nb_3_O_7_(OH) under sonication. After 30 min of sonication, the vessel was capped and transferred to the microwave reactor (CEM Discover, 220 W, 220 psi, *T* = 150 °C, 20 min). After centrifugation and being washed with deionized water, the sample was placed into a vacuum oven at *T* = 70 °C for 24 h. The dried Pd@Nb_3_O_7_(OH) sample was then treated at *T* = 120 °C in air for 24 h to obtain the final Pd@Nb_2_O_5_ nanostructured samples ready for characterization and catalytic testing.

*Characterization*: Sample morphology was determined using a Hitachi H‐7000 transmission electron microscopy (TEM) at 100 kV. The scanning transmission electron microscopy (STEM), high angle annular dark field (HAADF) imaging, and electron energy‐loss spectroscopy (EELS) analysis were performed using an aberration‐corrected Nion UltraSTEM‐200, equipped with a cold‐field emission gun. The Nion UltraSTEM‐200 was operated at 200 kV when performing HAADF imaging and EELS mapping. EEL spectra were collected using a Gatan Enfinium Dual EELS spectrometer. Principal component analysis (PCA) was performed on the EELS data to remove random noise.^44^

Powder X‐ray diffraction (PXRD) was performed on a Bruker D2‐Phaser X‐ray diffractometer, using Cu Kα radiation at 30 kV.

The surface area of each sample was determined through volumetric nitrogen adsorption at 77 K using a Quantachrome Autosorb‐1‐C and calculated using Brunauer‐Emmet‐Teller (BET) theory. Samples were outgassed overnight at 80 °C prior to being analyzed.

Diffuse reflectance spectra of the samples were measured using a Lambda 1050 UV/VIS/NIR spectrometer from Perkin Elmer equipped with an integrating sphere with a diameter of 150 mm. The film samples were prepared by drop‐casting aqueous dispersions of Pd@Nb_2_O_5_, Nb_3_O_7_(OH), and Nb_2_O_5_ samples onto borosilicate glass microfiber filters.

X‐ray photoelectron spectroscopy (XPS) was performed using a Perkin Elmer Phi 5500 ESCA spectrometer. The spectrometer uses an Al Kα X‐ray source to generate X‐rays with an energy of 1486.7 eV. The samples used in XPS analyses were prepared by drop‐casting aqueous dispersions of Pd@Nb_2_O_5_ and Nb_2_O_5_ samples onto p‐doped Si(100) wafers. All measurements were conducted in an ultrahigh vacuum chamber with a base pressure of 1 × 10^−9^ Torr. Data analyses were performed using the Multipak program and all binding energies were referenced to the NIST‐XPS database and the Handbook of X‐ray Photoelectron Spectroscopy.^35,36^

CO_2_ adsorption capacity was determined using thermogravimetric analysis (Discovery TGA, TA Instruments) with the following procedure. Each sample (≈5–10 mg) was first heated at a rate of 10 °C min^−1^ to a final temperature of 120 °C, under a nitrogen flow of 100 mL min^−1^. This temperature and nitrogen flow was maintained for 1 h to remove any adsorbed moisture. The gas flow was then switched to carbon dioxide, maintaining the same flow rate and temperature for 2 h. The observed weight gain between the N_2_ and CO_2_ gas streams was directly used to calculate CO_2_ capacity, and normalized against the surface area of each sample.

TGA was performed to determine whether or not carbon deposits reside on the used catalyst.^32^ These experiments were carried out using a Discovery TGA (TA Instruments). A catalyst film sample was prepared on frosted glass substrates (Erie Scientific) and tested for a duration of 40 h under testing conditions. After the catalytic tests, the “used” sample was scraped off the frosted glass substrate for TGA measurements. Two catalyst samples, one fresh sample and one used sample, were each heated under flowing CO_2_, at a rate of 100 mL min^−1^. The samples were heated at a rate of 1 °C min^−1^ to 700 °C. The same TGA experiments were also performed using air since it is known to efficiently remove carbon deposits.

The Raman spectra were obtained using the Horiba Jobin Yvon confocal system; namely a LabRAM HR 800 spectrometer equipped with a continuous wave HeNe laser (*λ* = 633 nm, with *r* = 400 nm) and a CW diode laser (*λ* = 785 nm, with *r* = 500 nm). The maximum laser power available using the HeNe laser was 12 mW. The samples were illuminated using a 100× objective. For the Raman spectra, which were obtained as a function of laser power, the exposure time varied from 1 to 40 s, with averaging over 4 spectra depending on the power used. The exposure time was increased as the incident power was reduced to maintain a signal to background ratio conducive to accurate peak fitting. Prior to fitting the Raman peaks, the spectra were identified and isolated from the measured data by subtracting a polynomial curve fit to the background signal. The stokes/anti‐stokes spectra were obtained using the 785 nm laser with a power of 24 mW. The samples for Raman measurements were prepared by drop‐casting aqueous dispersions of Pd@Nb_2_O_5_ and Nb_2_O_5_ samples onto borosilicate glass microfiber filters.

Electron paramagnetic resonance (EPR) measurements were performed at 20 K using a Bruker ECS‐EMX X‐band EPR spectrometer equipped with an ER4119HS cavity. An Oxford ESR 900 helium cryostat controlled by an Oxford ITC503 temperature controller was utilized. Typical operating parameters were as follows: microwave frequency 9.381799030 Ghz, microwave power 21.63 mW, modulation amplitude 4 G, sweep width 512 G centered at 3455.45 G, time constant 0.01 ms, total sweep time 512 s, 4096 points were acquired. The EPR analysis was applied to nanostructured Nb_2_O_5_ samples, which were sealed in the EPR tubes in the glove‐box under a N_2_ gas atmosphere.

*Gas‐Phase Catalytic Measurements*: For gas‐phase photothermal catalytic testing, samples were prepared by drop casting nanostuctured Nb_2_O_5_ and Pd@Nb_2_O_5_ from an aqueous dispersion onto binder free borosilicate glass microfiber filters having an area of ≈2.2 cm^2^ (Whatman, GF/F, 0.7 μm).

The gas‐phase photothermal catalytic measurements were conducted in a custom‐built 1.8 mL stainless steel batch reactor with a fused silica view port sealed with a Viton O‐ring. The reactor was evacuated using an Alcatel dry pump prior to being purged with H_2_ (99.9995%) at a flow rate of 20 mL min^−1^. After purging, the reactor was infiltrated with H_2_ and CO_2_ gas at a 1:1 pressure ratio to a total pressure of 27 psi prior to being sealed. The pressure inside the reactor was monitored during the reaction using an Omega PX309 pressure transducer. Reactors were irradiated with a 300 W Xe lamp for a duration of 3 h. The spectral output from the 300 W Xe lamp was measured using a StellarNet Inc spectrophotometer and the power of the incident irradiation was measured using a Spectra‐Physics Power meter (model 407A). The radiant power absorbed by the sample for each run was determined using the equation $P=\int P_{\lambda }\cdot t_{\lambda }\cdot a_{\lambda }\;d\lambda$; where *P_λ_* and *t*_λ_, are the power emitted by the Xe lamp and the fraction of light transmitted through the quartz window as well as any filters used in the measurement, respectively. The light absorbed by the sample was calculated using the equation *a_λ_ = 1 – R_λ_*, where *R_λ_* is the diffuse reflectance measured at wavelength *λ*. The reactors were not connected to a heating source for the photothermal tests, although the reactor temperature increased to 50 °C, over the 3 h duration of the test due to the heat generated from the incident irradiation (see Figure S14 in the Supporting Information). For thermal tests in the dark, the reactor temperature was controlled by an OMEGA temperature controller combined with a thermocouple placed in the reactor. Product gases were analyzed with a flame ionization detector (FID) installed in a SRI‐8610 gas chromatograph (GC) with a 6′ Haysep D column. Isotope tracing experiments were performed using ^13^CO_2_ (99.9 at%; Sigma Aldrich). The reactor was evacuated prior to being injected with ^13^CO_2_ followed by H_2_. Isotopically labeled product gases were measured using an Agilent 7890A gas chromatographic mass spectrometer (GC‐MS) with a 60 m GS‐Carbonplot column fed to the mass spectrometer.

## Supporting information

As a service to our authors and readers, this journal provides supporting information supplied by the authors. Such materials are peer reviewed and may be re‐organized for online delivery, but are not copy‐edited or typeset. Technical support issues arising from supporting information (other than missing files) should be addressed to the authors.

Supplementary
