# Supplementary material for: Visible and Near‐Infrared Photothermal Catalyzed Hydrogenation of Gaseous CO2 over Nanostructured Pd@Nb2O5
Source: Adv Sci (Weinh). 2016 Jul 5;3(10):1600189. doi: 10.1002/advs.201600189 (PMC5095794; doi:10.1002/advs.201600189)
Supplement: Supplementary file 1 — Supplementary [file ADVS-3-0a-s001.pdf]

Copyright WILEY-VCH Verlag GmbH & Co. KGaA, 69469 Weinheim, Germany, 2016.

ADVANCED SCIENCE Open Access

# Supporting Information

for *Adv. Sci.,* DOI: 10.1002/advs.201600189

# Visible and Near-Infrared Photothermal Catalyzed Hydrogenation of Gaseous $CO_2$ over Nanostructured Pd@$Nb_2O_5$

*Jia Jia, Paul G. O'Brien, Le He, Qiao Qiao, Laura M. Reyes, Timothy E. Burrow, Yuchan Dong, Kristine Liao, Maria Varela, Stephen J. Pennycook, Mohamad Hmadeh, Amr S. Helmy, Nazir P. Kherani, Doug D. Perovic, and Geoffrey A. Ozin**

Copyright WILEY-VCH Verlag GmbH & Co. KGaA, 69469 Weinheim, Germany, 2016.

# Supporting Information

DOI: 10.1002/advs.201600189

**Title: Visible and Near Infrared Photothermal Catalyzed Hydrogenation of Gaseous $CO_2$ over Nanostructured $Pd@Nb_2O_5$**

*Jia Jia,[†] Paul G. O'Brien,[†] Le He, Qiao Qiao, Teng Fei, Laura M. Reyes, Timothy E. Burrow, Yuchan Dong, Kristine Liao, Maria Varela, Stephen J. Pennycook, Mohamad Hmadeh, Amr S. Helmy, Nazir P. Kherani, Doug D. Perovic, and Geoffrey A. Ozin **

**Figure S1** (a) HAADF image of the $Nb_3O_7(OH)$ nanorods at low magnification. (b) HAADF image taken from a nanorod in its [001] direction. The preferred growth direction (longitudinal) of the nanorod is [010]. (c) FFT of (b) showing (200), (110) and (020) spots. (d) Atomic model indicated by the red rectangle in (b), where the dotted blue rectangle indicates a $Nb_3O_7(OH)$ unit cell.

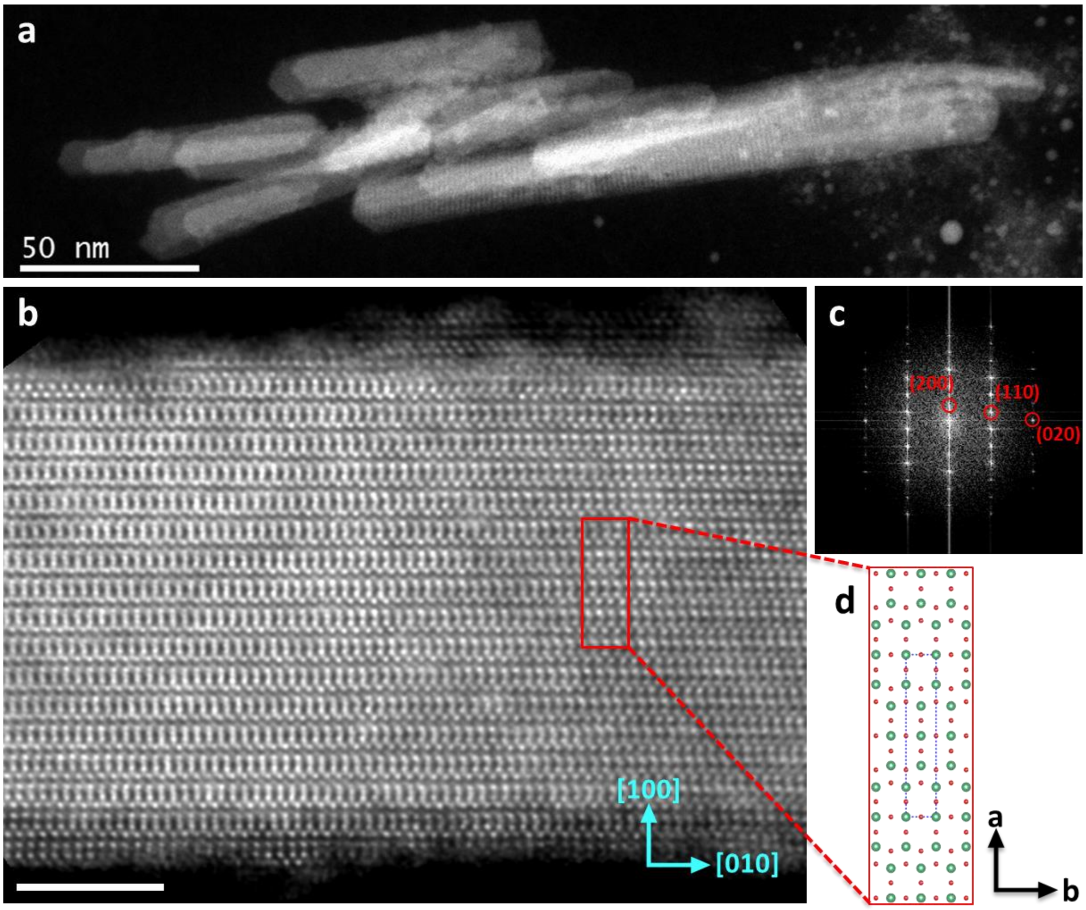

**Figure S2.** Powder X-ray diffraction patterns of $Nb_2O_5$, $Nb_3O_7(OH)$, 0.5% $Pd@Nb_2O_5$ (after pre-treatment) and 0.5% $Pd@Nb_3O_7(OH)$ (before pre-treatment).

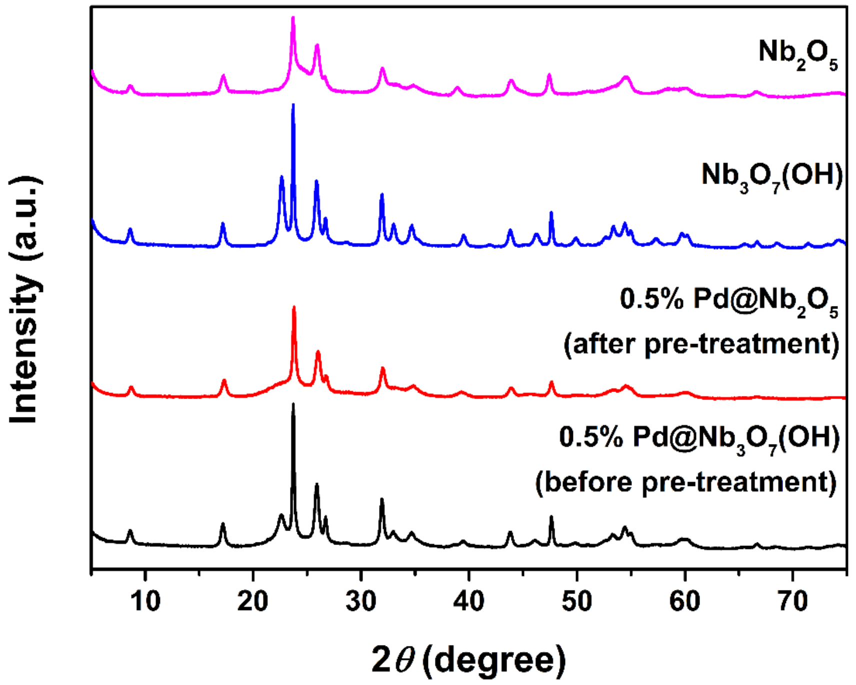

**Figure S3.** TEM images of (a) $Nb_3O_7(OH)$ nanorods and (b) $Nb_2O_5$ nanorods.

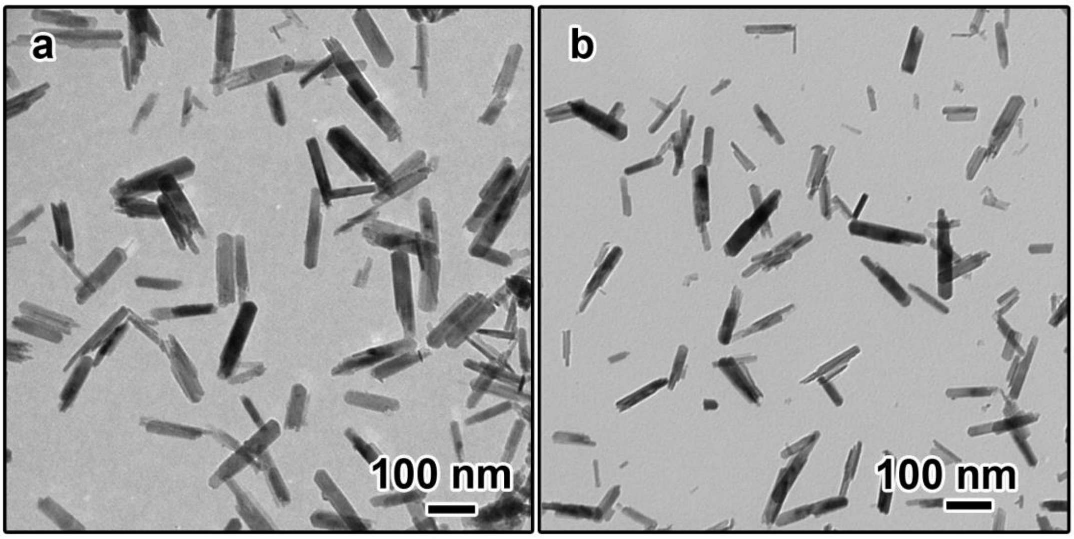

**Figure S4.** High resolution XPS spectra of the (a) Nb3d, (b) O1s and (c, d) Pd3d regions taken on $Nb_2O_5$, Pd@$Nb_2O_5$, 0.5% Pd@$Nb_2O_5$ and 1% Pd@$Nb_2O_5$, respectively.

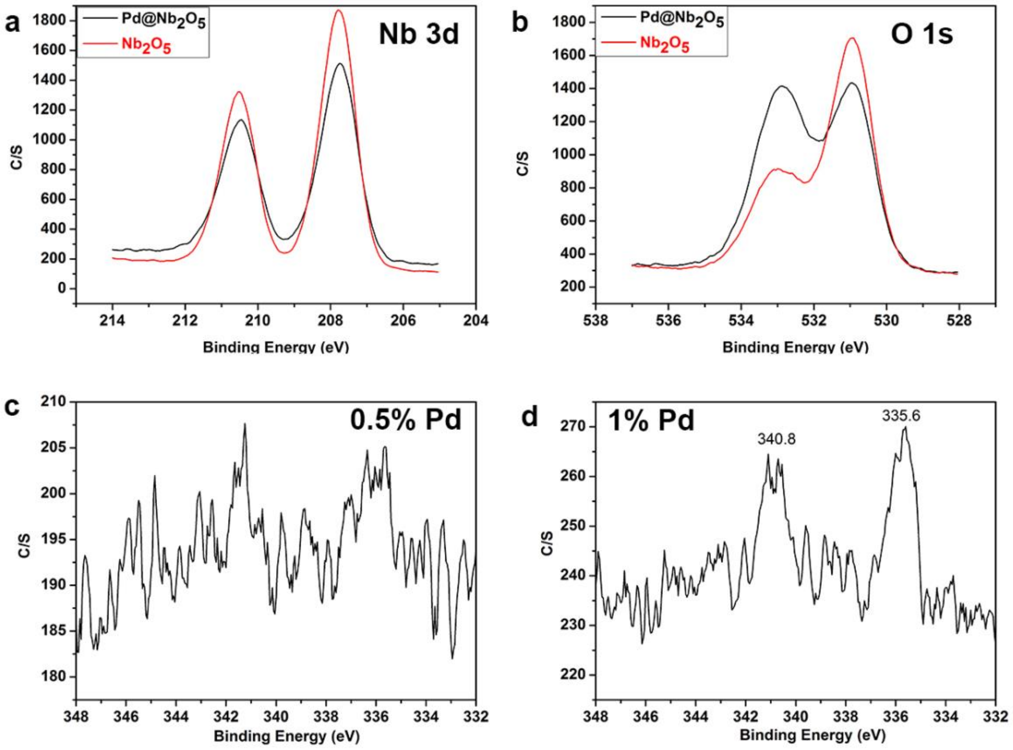

**Figure S5.** Estimation of the electronic band gap of $Nb_2O_5$. By using a modified Kubelka-Munk function, $(F(R)*h\nu)^n$ is plotted as a function of photon energy for $Nb_2O_5$ where $F(R) = (1-R)^2/2R$. Here R is the diffuse reflectance of the films loaded onto the borosilicate sample supports and n was set to 2 for $Nb_2O_5$. The linear portion of the plot was extrapolated and its intercept with the abscissa is considered to be the band-gap.

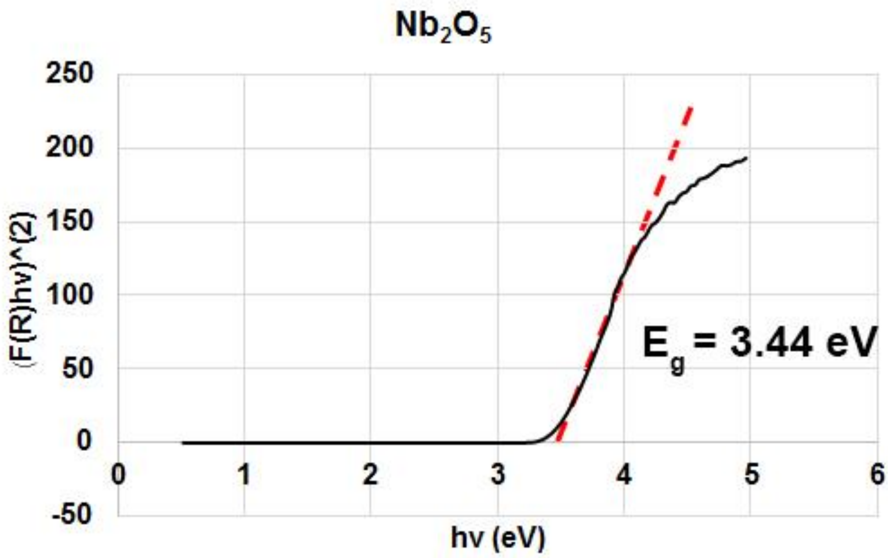

**Figure S6.** XPS Spectra of $Nb_2O_5$ (a) secondary electron cut-off region. (b) Valence band region (3.3 eV is relative to the work function.)

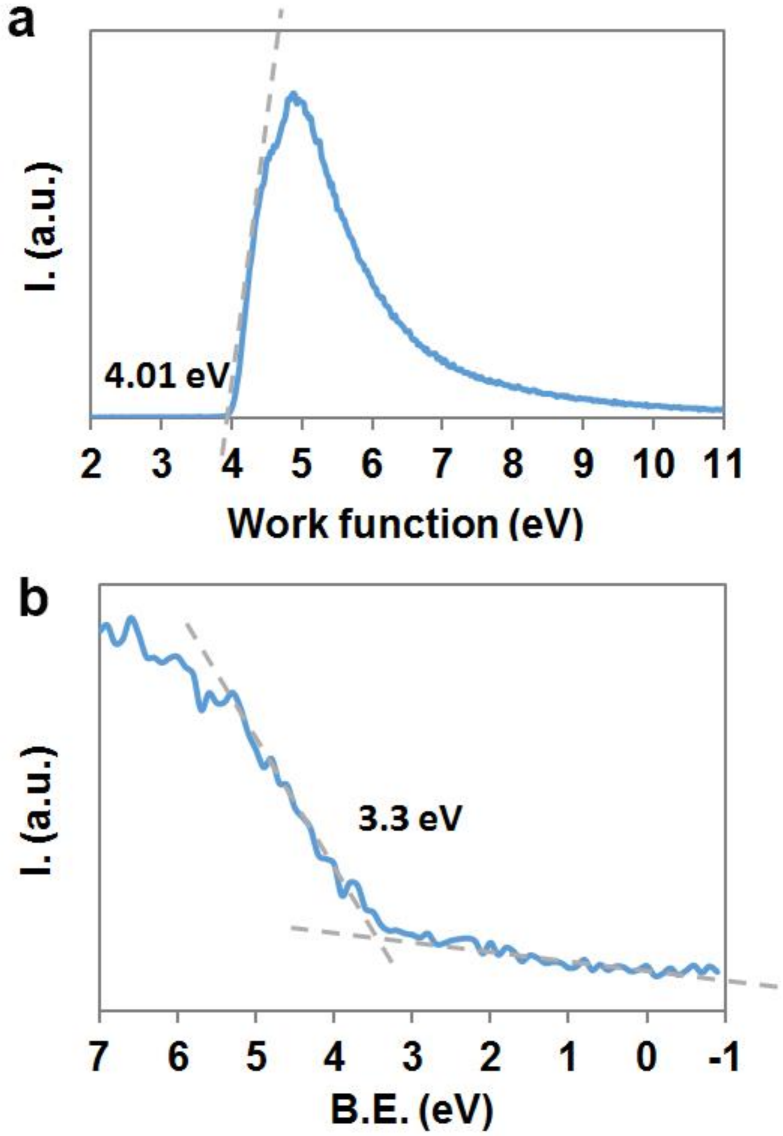

**Figure S7.** Mass spectrometry of photo-thermally generated $^{13}CO$ from $^{13}CO_2$. The 28 AMU mass fragment peak at approximately 1.32 min corresponds to $N_2$ and the 29 AMU mass fragment peak at approximately 1.345 min corresponds to $^{13}CO$. The fact that there is no peak in the vicinity of 1.345 min retention time for the 28 AMU curve shows that there is no $^{12}CO$ in the products that could have been generated from sources of adventitious $^{12}C$.

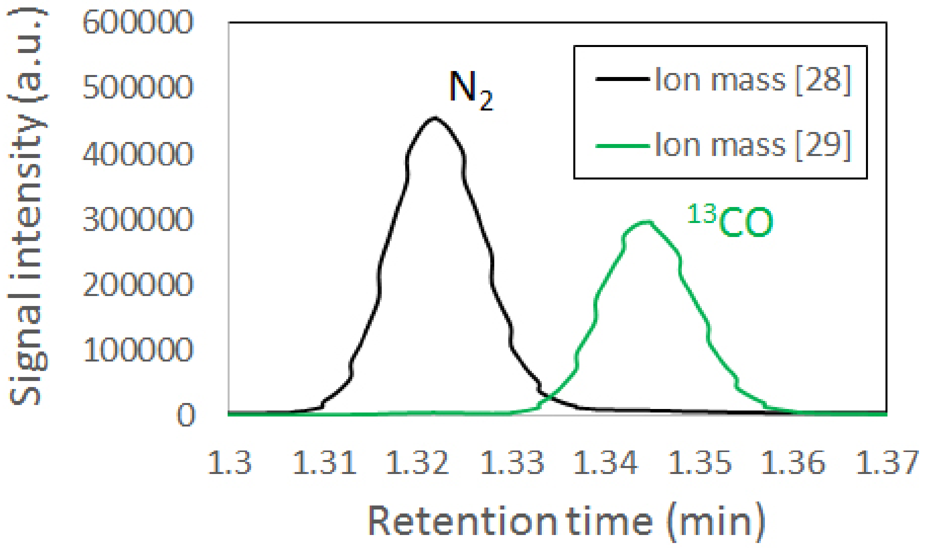

**Figure S8.** TEM image of Pd@$Nb_2O_5$ after gas-phase catalytic testing.

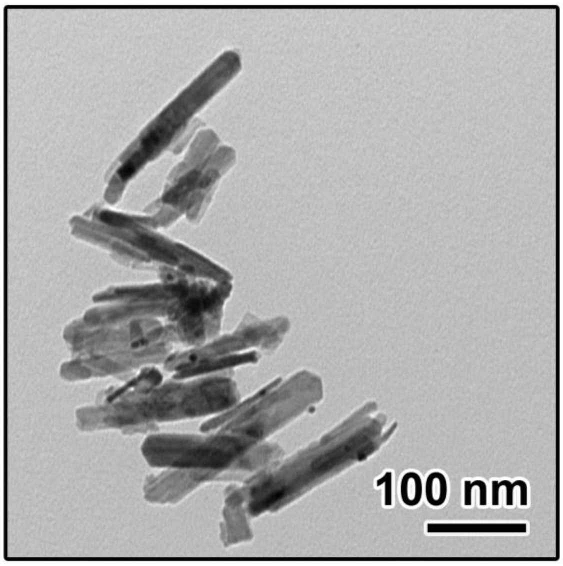

**Figure S9.** CO production rate from $CO_2$ in the presence and absence of $H_2$ over Pd@$Nb_2O_5$.

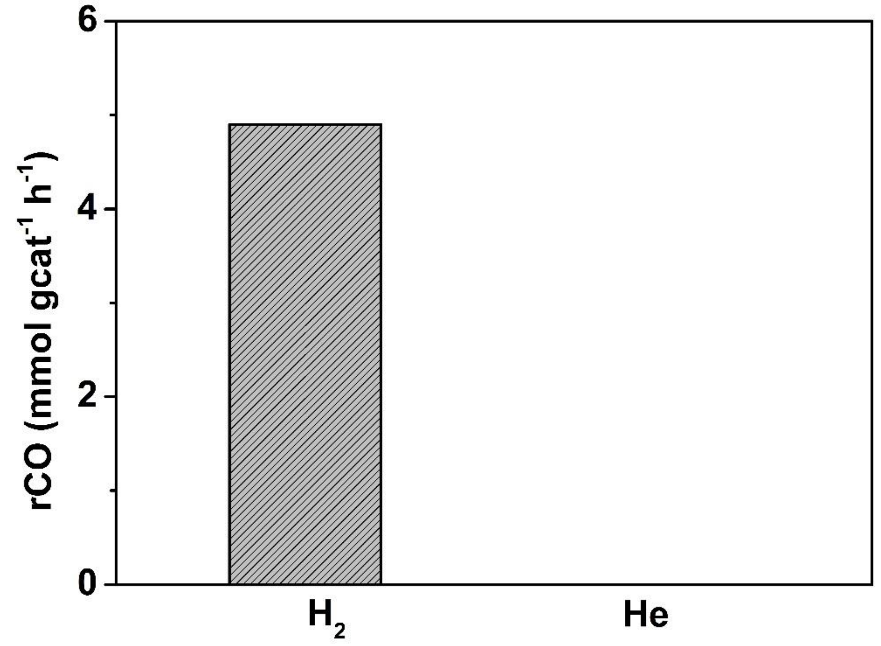

**Figure S10.** Thermogravimetric analysis (TGA) and differential thermogravimetric analysis of the fresh catalyst and used catalyst after catalytic reaction tests of 40 h

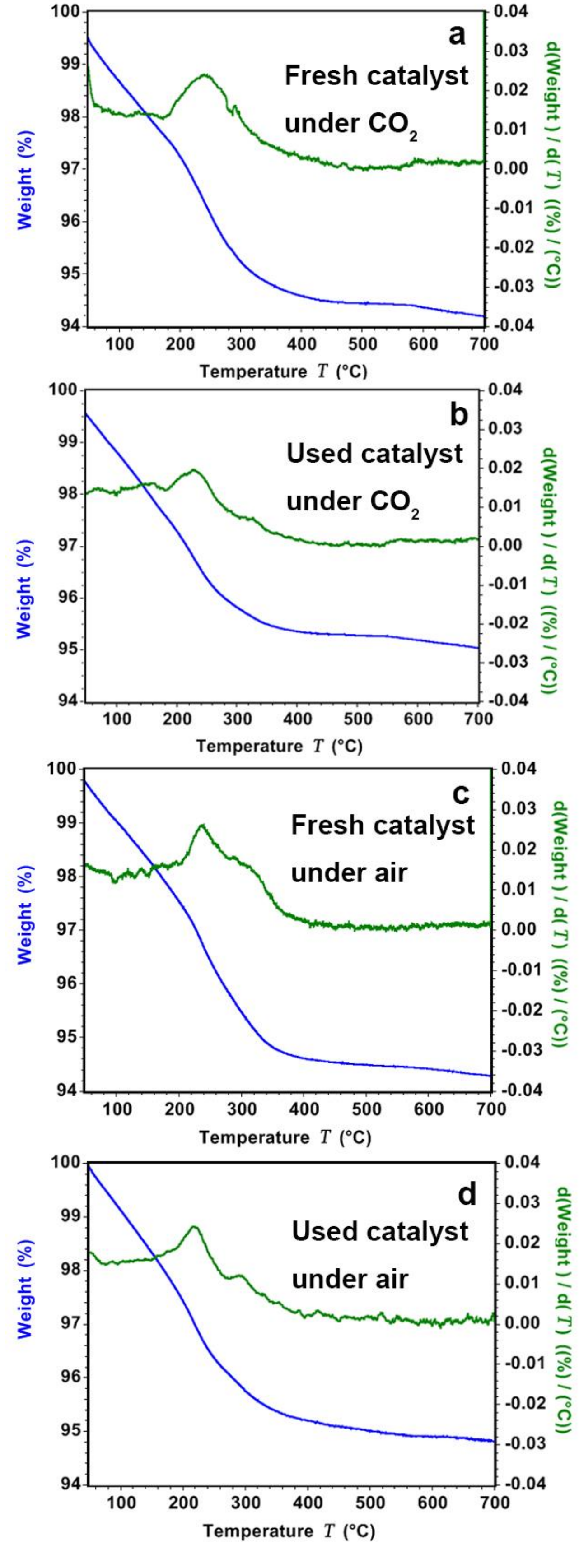

**Figure S11.** Dependence of the Raman frequency from 100 $cm^{-1}$ to 1500 $cm^{-1}$ for νNb=O stretching vibrations of a) Pd@$Nb_2O_5$ and b) $Nb_2O_5$ nanorods at different power levels.

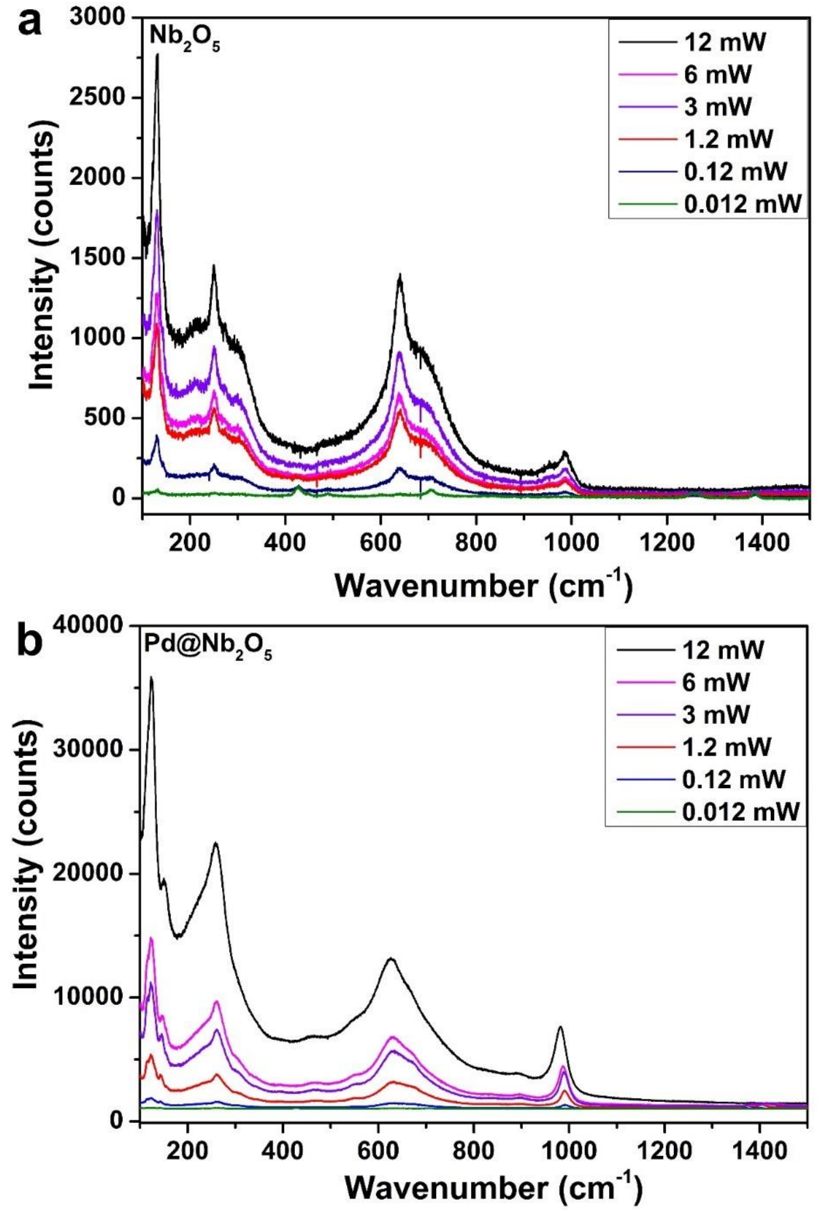

**Figure S12.** Selected wavelength Stokes and anti-Stokes Raman spectra between 500 $cm^{-1}$ and 900 $cm^{-1}$ with representative deconvolution of the spectra analyzed by LabSpec software for a) Pd@$Nb_2O_5$ Stokes spectra; b) Pd@$Nb_2O_5$ anti-Stokes spectra; c) $Nb_2O_5$ Stokes spectra and d) $Nb_2O_5$ anti-Stokes spectra.

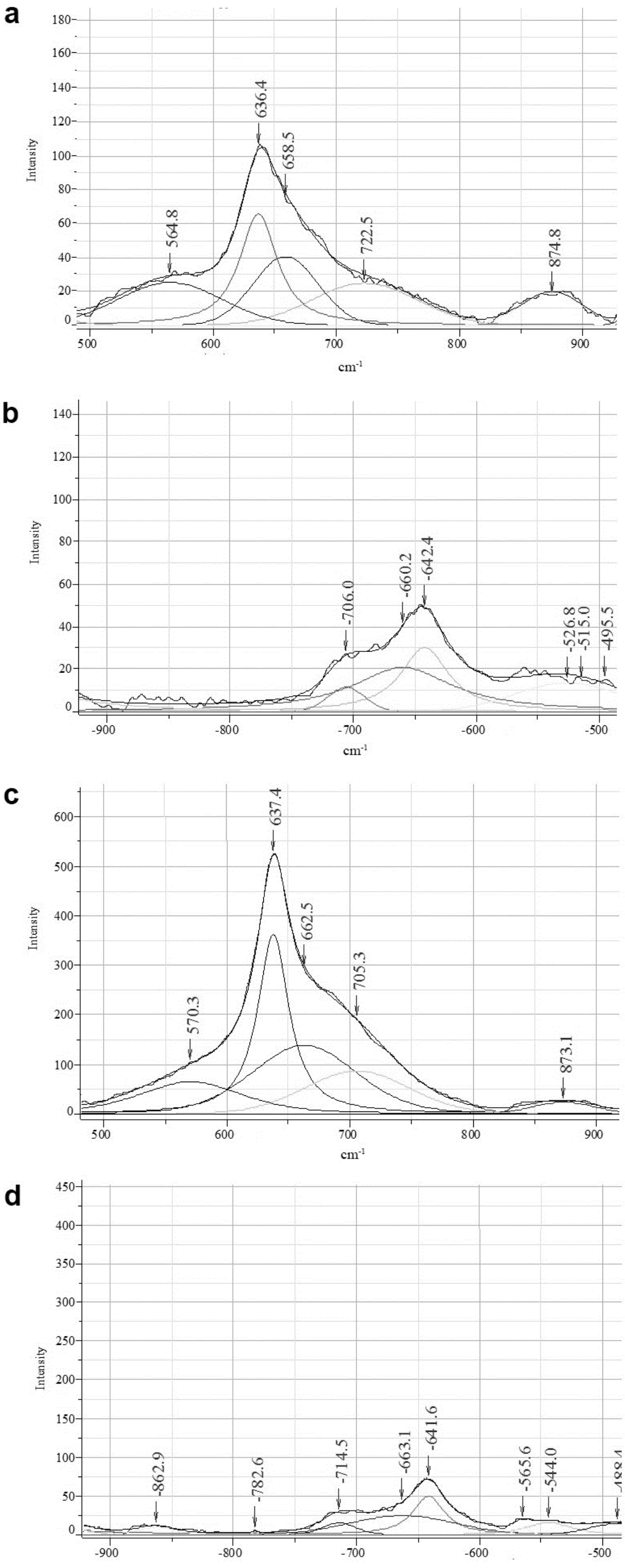

**Figure S13.** EPR spectra of $Nb_2O_5$ before (black) and after (red) being illuminated under a 300 W Xe lamp for 2 hours, recorded at 20 K.

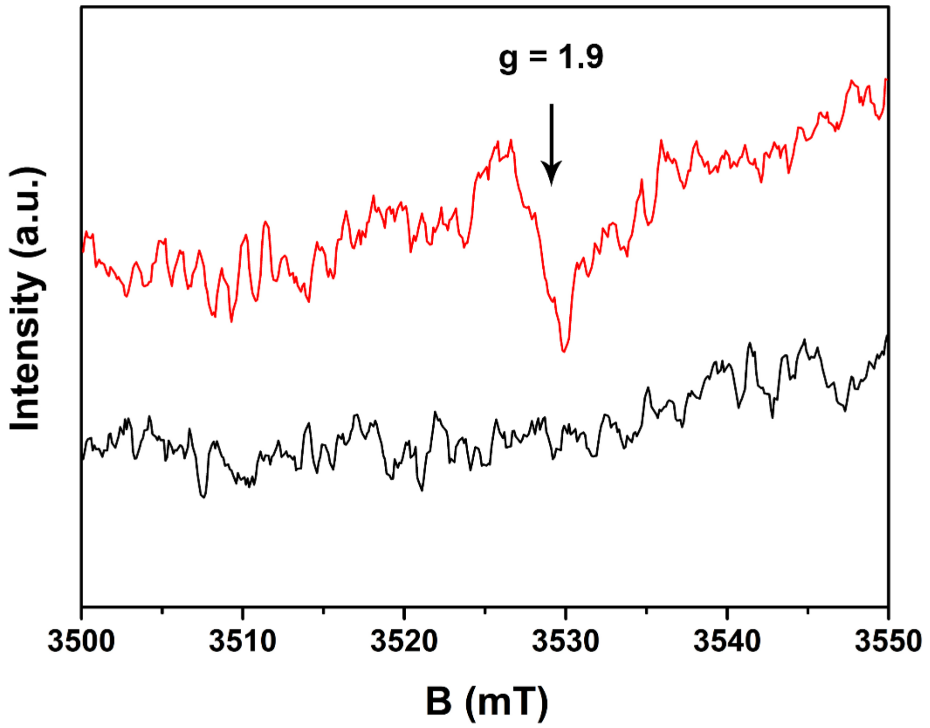

**Figure S14.** The photoreactor temperature as a function of time over the duration of a photothermal test. The temperature inside the reactor is 49.7 °C at the 3 h point of the test. The temperature of the sample is much greater than that of the reactor due to the photothermal effect.

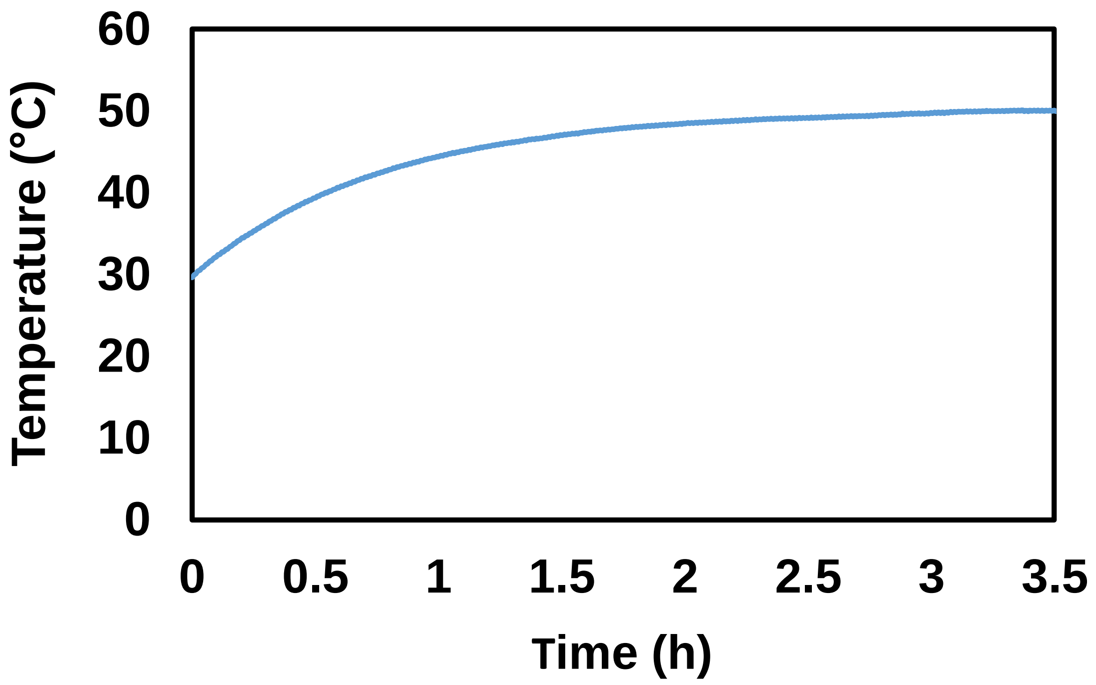

**Table S1.** Estimation of reaction temperatures over Pd@$Nb_2O_5$ under irradiation from the 300 W Xe lamp using different cut-off filters for batch reactions A through F and without any filter at different light intensities for batch reactions I through V.

| Sample | k (mmol $g^{-1}h^{-1}$) | T(K estimated) | T(°C estimated) |
|---|---|---|---|
| A(I) | 4.9 | 470 | 197 |
| B | 3.5 | 462 | 190 |
| C | 1.8 | 447 | 174 |
| D | 1.1 | 436 | 163 |
| E | 0.78 | 430 | 157 |
| F | 0.41 | 417 | 144 |
| II | 3.3 | 461 | 189 |
| III | 0.95 | 434 | 161 |
| IV | 0.63 | 426 | 153 |
| V | 0.56 | 423 | 150 |

**Table S2**. Summary of the Raman band frequency for the νNb=O stretching vibrations of Pd@$Nb_2O_5$ and $Nb_2O_5$ at different power levels using a 633 nm laser. Temperature estimation was performed using the slope of the dependence of the Raman band shift on temperature in prior work.[1]

| **Power levels (mW)** | **Pd@$Nb_2O_5$** | | | **$Nb_2O_5$** | | |
|---|---|---|---|---|---|---|
| | **Peak position ($cm^{-1}$)** | **Band shift ($cm^{-1}$)** | **Estimated T (K)** | **Peak position ($cm^{-1}$)** | **Band shift ($cm^{-1}$)** | **Estimated T (K)** |
| 12 | 981.5 | -10.5 | 777 | 985.7 | -1.5 | 368 |
| 6 | 986.9 | -5.1 | 531 | 986.3 | -0.9 | 341 |
| 3 | 989.3 | -2.7 | 422 | 986.6 | -0.6 | 327 |
| 1.2 | 990.5 | -1.5 | 368 | 986.9 | -0.3 | 314 |
| 0.12 | 991.1 | -0.9 | 341 | 987.2 | 0 | 300 |
| 0.012 | 992.0 | 0 | 300 | 987.2 | 0 | 300 |

**Table S3**. Estimation of the temperature of Pd@$Nb_2O_5$ and $Nb_2O_5$ under the power level of 24 mW using a laser pump wavelength of 785 nm. The signals of the Raman Stokes and anti-Stokes scattering at around 640 $cm^{-1}$ are obtained from the deconvolution of the spectra by LabSpec software, as shown in Figure S11. The estimation of temperatures is calculated using the ratio of Stokes signals to anti-Stokes signals and the band position of the Raman modes.

| **Sample** | **Stokes signal (S) area** | **anti-Stokes (AS) signal area** | **S/AS Ratio** | **ω ($cm^{-1}$)** | **T (K)** |
|---|---|---|---|---|---|
| Pd@$Nb_2O_5$ | 65.96 | 30.18 | 2.185 | 636.44 | 776 |
| $Nb_2O_5$ | 362.91 | 49.77 | 7.291 | 637.42 | 385 |

Reference

[1] S. Xie, E. Iglesia, A. T. Bell, *J. Phys. Chem. B* **2001**, 105, 5144.
